# Molecular evidence for cryptic species in the common slug eating snake *Duberrialutrixlutrix* (Squamata, Lamprophiidae) from South Africa

**DOI:** 10.3897/zookeys.838.32022

**Published:** 2019-04-15

**Authors:** Kyle Kulenkampff, Francois Van Zyl, Sebastian Klaus, Savel R. Daniels

**Affiliations:** 1 Department of Botany and Zoology, Private Bag X1, Stellenbosch University, Matieland, 7602, South Africa Stellenbosch University Stellenbosch South Africa; 2 Department of Ecology and Evolution, J. W. Goethe-University, Biologicum, Frankfurt am Main, Germany J. W. Goethe-University Frankfurt Germany

**Keywords:** snakes, Afrotropical, alpha taxonomy, phylogenetics, altitudinal barriers, southern Africa

## Abstract

We examined the impact of climatic fluctuations on the phylogeographic structure of the common slug eating snake (*Duberrialutrixlutrix*) throughout its distribution in South Africa. The evolutionary history within the taxon was examined using partial DNA sequence data for two mitochondrial genes (ND4 + cyt *b*) in combination with a nuclear locus (SPTBN1). Phylogenetic relationships were investigated for both the combined mtDNA and total evidence DNA sequence data. In addition, population and demographic analyses together with divergence time estimations were conducted on the combined mtDNA data. Topologies derived from the combined mtDNA analyses and the total evidence analyses were congruent and retrieved five statistically well-supported clades, suggesting that *Duberrial.lutrix* represents a species complex. The five clades were generally allopatric, separated by altitudinal barriers and characterised by the absence of shared mtDNA haplotypes suggesting long term isolation. Divergence time estimations indicate that the diversification within the *D.l.lutrix* species complex occurred during the Plio/Pleistocene as a result of climatic fluctuations and habitat shifts for the species. A taxonomic revision of the *D.l.lutrix* species complex may be required to delineate possible species boundaries.

## Introduction

Climatic oscillations are thought to be responsible for inducing dramatic impacts on the habitat and eco-physiological characteristics promoting cladogenesis (Hewitt 2000, 2004; Daniels et al. 2007, 2009; Barlow et al. 2013; Engelbrecht et al. 2013). The effects of climatic impacts on terrestrial biota vary considerably depending on latitude, longitude, habitat and the topographic heterogeneity of the environment (Hewitt 2000, 2004). Northern temperate continental areas experienced significant Pliocene/Pleistocene climatic changes whilst many biomes closer to the equator were reduced in size due to increased aridity and the expansion of arid environments (Hewitt 2000, 2004). These northern hemispherical climatic conditions resulted in noticeable recent cladogenesis for numerous species (Clark et al. 1999; Hewitt 2000, 2004; Lisiecki and Raymo 2007). In contrast, the impact of climatic changes on speciation in southern hemisphere terrestrial biota remains less studied, with reptiles being particularly neglected (Hewitt 2004; Barlow et al. 2013). Reptiles, as ectotherms, are particularly sensitive to temperature fluctuations and are thus ideal organisms with which to test the impact of climate ameliorations on phylogeographic patterning (Santos et al. 2012; Martínez-Freiría et al. 2015).

The serpent fauna of South Africa contains 116 species, of which 29 are endemic (Bates et al. 2014). There is a paucity of evolutionary studies of South African snakes, limiting our understanding of the patterns and processes that resulted in the contemporary population genetic structure. A phylogeographic study on the widespread African puff adder, *Bitisarietans*, revealed the presence of six refugial areas that existed during the last glacial maximum (LGM) occurring along the west coast, south west coast, southeast coast, in the northern regions, as well as two occurring in the central northern part of South Africa (Barlow et al. 2013). Declining temperatures and increased aridification associated with climatic oscillations are suspected to be causal to the isolation among the *B.arietans* clades (Barlow et al. 2013). One of southern Africa’s most widely distributed snake species is the Common Slug Eater, *Duberrialutrixlutrix* (Branch 1998; Spawls et al. 2002; Uetz et al. 2019). *Duberrialutrixlutrix* is a molluscivorous, viviparous, non-venomous, small-bodied snake (Branch 1998). The species occurs in savannah, grassland, coastal bushveld and fynbos habitats (Branch 1998; Bates et al. 2014) where it prefers mesic areas (Branch 1998; Rabiega 2013; Bates et al. 2014). Four *Duberria* species (*D.lutrix*, *D.rhodesiana*, *D.shirana* and *D.variegata*) and five subspecies (*D.l.lutrix*, *D.l.abyssinica*, *D.l.atriventris*, *D.l.basilewskyi* and *D.l.currylindhali*, are currently recognised (Wallach et al. 2014; Uetz et al. 2019). The taxonomic status of these subspecies remains dubious, and some of these subspecies likely represent full species. *Duberrial.lutrix* is the only subspecies that occurs in South Africa, ranging in distribution from the coastal belt fringes of the Western Cape, Eastern Cape, KwaZulu-Natal, Gauteng, Mpumalanga and Limpopo provinces while allopatric, presumably relictual populations, are restricted to the interior in the Klein Karoo and Free State Province of South Africa (Branch 1998, 2002; Rabiega 2013; Bates et al. 2014).

**Figure 1.**
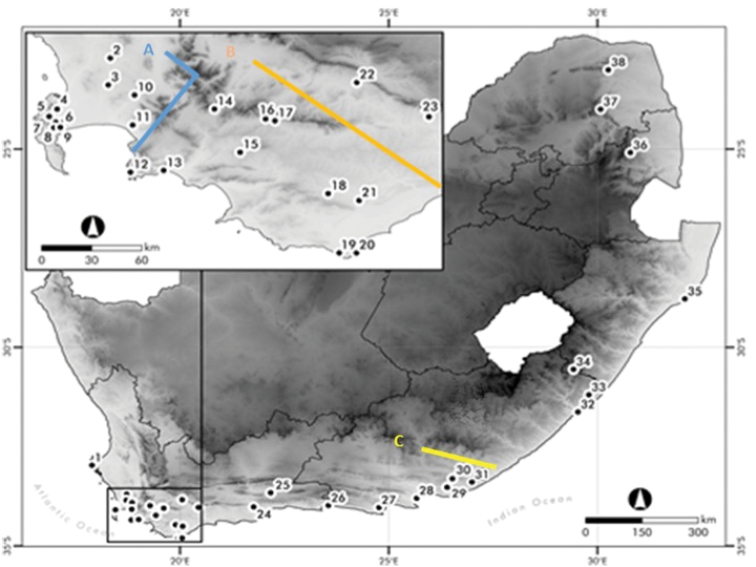
Known biogeographic breaks for co-distributed animal species in South Africa, including the **A** Hottentots Holland Mountains (blue) **B** The Breede River Valley (red) and **C** the Bedford gap (Yellow). The *Duberrial.lutrix* localities sampled throughout South Africa during the present study. Locality numbers correspond to the sample site numbers in Table 1.

The distribution range of *Duberrial.lutrix* in South Africa is bisected by several large mountain ranges, including the Cape Fold Mountains and the Great Drakensberg escarpment (Partridge and Maud 1987, 2000; Partridge 1997) and low-lying xeric corridors. The habitat availability of the species in the Western Cape Province would have undergone significant climatic shifts from mesic conditions in the Miocene to enhanced arid conditions during the Pliocene/Pleistocene, possibly impacting habitat availability for this mesophylic species and the contraction of populations to high-lying mountainous refugia (Daniels et al. 2007; Engelbrecht et al. 2013; Diedericks and Daniels 2014). Xeric areas could potentially act as dispersal barriers for a small-bodied snake species with a preference for mesic environments (Hewitt 2004). The distribution of *D.l.lutrix* overlaps with several phylogeographic breaks for co-distributed lizard species (Fig. 1) (Daniels et al. 2007; Engelbrecht et al. 2013; Diedericks and Daniels 2014). A xeric biogeographic barrier known as the Bedford gap exists which separates the eastern and south-eastern part of the Cape Floristic Region (CFR) between East London and Port Elizabeth (Lawes 1990). This biogeographic gap is characterised by the intrusion of a sub-desert biome resulting in a semi-arid climate and is thought to have existed since the late Pliocene, resulting in unfavourable habitat for *D.l.lutrix* (Lawes 1990; Lawes et al. 2007).

Considering the results observed in phylogeographic studies of other co-distributed reptile species, we postulate that *D.l.lutrix* exhibits similar patterns of genetic differentiation across its distribution in South Africa. The objective of the present study is twofold. First, to examine the phylogeographic relationships within *D.l.lutrix* in South Africa, and to explore the possible impact of climatic changes on the phylogeographic patterning of the species. Second, to examine the presence of possible cryptic lineages within the taxon. Firstly, we hypothesize that climatic induced evolutionary changes during the Plio/Pleistocene promoted cladogenesis in the species. Secondly, we hypothesize that discrete lineages are present within *D.l.lutrix*.

## Methods and materials

### Sample collection

Road killed specimens or tissue of *Duberrial.lutrix* were obtained from the South African National Biodiversity Institute tissue bank (SANBI – Cape Town, South Africa) and from several private collections and road kills. Road killed specimens or tissue samples were preserved in absolute ethanol and refrigerated at 4 °C. A total of 87 *D.l.lutrix* specimens were collected from 38 localities across South Africa, covering most of the subspecies distribution range (Fig. 1, Table 1). An additional *Duberria* specimen from Uganda was donated by the Californian Academy of Sciences (CAS), USA. Specimens collected during the present study were deposited in the Port Elizabeth Museum Reptile Collection (PEM R), Eastern Cape Province, South Africa (Table 1).

**Table 1.** A list representing the locality, number of samples (*N*), province, coordinates and number of individuals selected for each gene of *Duberrialutrixlutrix* samples collected. The sample site number corresponds to Fig. 1. An additional sample from Vidal et al. (2008) is indicated with a ‡. PEM = Port Elizabeth Museum, SANBI = South African National Biodiversity Institute.

Sample number	Locality	*N*	Museum / SANBI tissue no	Province	Coordinates	Genbank Accesion numbers		
						ND4	cyt b	SPTBN1
1	Jacobs Bay	1	PEM R22493	Western Cape	32°58'8.27"S, 17°53'27.35"E	MK518189	MK518103	MK518271
2	Klipheuwel	2	unaccessioned	Western Cape	33°41'46.42"S, 18°43'26.97"E	MK518249–50	MK518108–09	MK518274
3	Kraaifontein	1	unaccessioned	Western Cape	33°50'57.39"S, 18°42'46.34"E	MK518200	MK518117	No Sequence
4	Kirstenbosch	3	SANBI 11300, 1450, 2785	Western Cape	33°59'10.89"S, 18°26'12.16"E	MK518190–92	MK518104–06	MK518272
5	Vlakkenberg	1	SANBI 4547	Western Cape	34°1'38.81"S, 18°23'37.87"E	MK518180	MK518093	MK518287
6	Bergvliet	1	unaccessioned	Western Cape	34°3'27.89"S, 18°27'6.26"E	MK518177	MK518089	MK518261
7	Tokai	1	SANBI 4545	Western Cape	34°3'37.40"S, 18°25'44.61"E	MK518232	MK518157	MK518285
8	Silvermine	1	SANBI 4550	Western Cape	34°5'29.69"S, 18°25'12.32"E	MK518219	MK518136	No Sequence
9	Lakeside	1	SANBI 1703	Western Cape	34°5'22.09"S, 18°27'14.02"E	MK518202	MK518119	MK518277
10	Stellenbosch	4	PEM R22494-97	Western Cape	33°54'23.90"S, 18°51'17.47"E	MK518227–31	MK518145–48	MK518282
11	Somerset West	8	PEM R22498-505	Western Cape	34°4'36.34"S, 18°50'40.88"E	MK518220–26, 51	MK518137–44	MK518281
12	Pringle Bay	4	PEM R22506-509	Western Cape	34°20'43.89"S, 18°49'58.08"E	MK518213–16	MK518130–33	No Sequence
13	Kleinmond	1	unaccessioned	Western Cape	34°20'6.03"S, 19°0'44.03"E	MK518248	MK518107	MK518273
14	Villiersdorp	5	PEM R22510-514	Western Cape	33°59'8.86"S, 19°17'10.18"E	MK518234–38	MK518159–63	MK518286
15	Caledon	1	PEM R22515	Western Cape	34°13'51.55"S, 19°25'31.35"E	MK518178	MK518091	MK518263
16	Genadendal	1	PEM R22516	Western Cape	34°2'29.91"S, 19°33'45.08"E	MK518181	MK518094	MK518265
17	Greyton	3	PEM R22517-519	Western Cape	34°3'8.18"S, 19°36'47.06"E	MK518182–84	MK518096–98	MK518266
18	Napier	6	PEM R22520-525	Western Cape	34°27'59.60"S, 19°53'59.71"E	MK518203–08	MK518120–25	MK518278
19	Agulhas	3	unaccessioned	Western Cape	34°48'11.21"S, 19°57'35.67"E	MK518243–45	MK518078–80	MK518259
20	Struis Bay	1	unaccessioned	Western Cape	34°45'42"S, 20°2'26.90"E	MK518252	MK518149	MK518283
21	Bredasdorp	1	unaccessioned	Western Cape	34°30'20.84"S, 20°4'2.12"E	MK518246	MK518090	MK518262
22	Ashton	8	PEM R22526-533	Western Cape	33°50'4.56"S, 20°3'17.09"E	MK518169–76	MK518081–88	MK518260
23	Swellendam	7	PEM R22524-540	Western Cape	34°1'45.81"S, 20°26'42.59"E	MK518253–58	MK518150–56	MK518284
24	Herbertsdale	1	SANBI 10847	Western Cape	34°1'1.25"S, 21°46'0.25"E	MK518185	MK518099	MK518267
25	Oudtshoorn	1	SANBI 2879	Western Cape	33°39'37.07"S, 22°10'24.69"E	MK518210	MK518127	MK518280
26	Natures Valley	1	SANBI 4558	Western Cape	33°58'50.40"S, 23°33'22.79"E	MK518209	MK518126	MK518279
27	Humansdorp	1	SANBI 8108	Eastern Cape	34°1'59.61"S, 24°45'59.39"E	MK518188	MK518102	MK518270
28	Port Elizabeth‡	1	Vidal et al. (2008)	Eastern Cape	33°47'52.79"S, 25°40'18.74"E	FJ404356	FJ494305	No Sequence
29	Hope Fountain	1	SANBI 4474	Eastern Cape	33°31'27.29"S, 26°24'3.91"E	MK518187	MK518101	MK518269
30	Grahamstown	1	unaccessioned	Eastern Cape	33°17'59.89"S, 26°31'37.88"E	MK518247	MK518095	No Sequence
31	Port Alfred	1	SANBI 400	Eastern Cape	33°35'35.60"S, 26°53'3.55"E	MK518211	MK518128	No Sequence
32	Port St John	1	SANBI 12180	Eastern Cape	31°37'45.67"S, 29°32'11.45"E	MK518212	MK518129	No Sequence
33	Kwancele	1	PEM R22541	Eastern Cape	31°11'41.74"S, 29°47'46.19"E	MK518201	MK518188	MK518276
34	Kokstad	7	PEM R22542-548	KwaZulu-Natal	30°33'14.70"S, 29°25'38.49"E	MK518193–99	MK518110–16	MK518275
35	High Water	1	SANBI 5307	KwaZulu-Natal	28°46'52.79"S, 32°5'54.60"E	MK518186	MK518100	MK518268
36	Sabie	2	PEM R 22549-550	Mpumalanga	25°5'19.90"S, 30°47'31.58"E	MK518217–218	MK518134–35	No Sequence
37	Wolkberg	1	unaccessioned	Limpopo	24°00'04.3"S, 30°04'37.4"E	MK518242	MK518164	MK518288
38	Entabeni	1	SANBI 1942	Limpopo	23°0'27.66"S, 30°15'49.80"E	MK518179	MK518092	MK518264
39		1	CAS 204338	Uganda	–	MK518233	MK518158	No Sequence

### DNA extraction, PCR amplification and sequencing

DNA was extracted from ethanol preserved muscle or liver tissue biopsies. A MacheryNagel DNA extraction kit was used for the DNA extraction, following the manufacturer’s protocol. Extracted DNA was stored at -20 °C until needed for the polymerase chain reaction (PCR). Three gene regions were targeted using the PCR, these included two mitochondrial (mtDNA) loci: nicotinamide adenine dinucleotide dehydrogenase subunit 4 (ND4) using the primer pairs listed in Arévalo et al. (1994) and Barlow et al. (2013) respectively; while for cytochrome b (cyt *b*) the primer pairs listed in Burbrink et al. (2000) and Ruane et al. (2015) were used; for the nuclear locus, β-spectrin nonerythrocytic intron 1 (SPTBN1) the primers pairs listed in Ruane et al. (2014) were used (see Table 2 for details of the primer pair combinations). The ND4 and cyt *b* loci have been extensively used in snake phylogeographic studies, while the nuDNA locus has been demonstrated to be a variable nuclear marker in other snake species (Burbrink et al. 2000; Barlow et al. 2013; Ruane et al. 2014, 2015). All specimens were sequenced for the two mtDNA loci, while a single sample per locality was sequenced for the nuDNA locus.

All PCR amplification was performed using standard protocols. PCR conditions were as followed: 94 °C for 4 min., 94 °C for 30 sec., the annealing temperature of the primers varied between 48 °C to 50 °C for 35 sec., 72 °C for 40 sec., for 35 cycles and a final extension at 72 °C for 10 min. A list of the six primer pairs used are provided in Table 2. PCR products were visualized using a 1% agarose gel that contained a 1% ethidium bromide solution. Following successful amplification of a locus, a BioFLUX gel purification kit was used to purify the amplicons, following the manufacturer’s protocol. The gel purified PCR amplicons were sequenced at the Central Analytical Facility (CAF), at Stellenbosch University, using an ABI 3700 automated DNA sequencer.

**Table 2.** List of the primer pairs and their respective reference used during the present study on *Duberrialutrixlutrix*.

Locus	Protein coding	Primer name and sequence	Primer reference
ND4	Yes	ND4: 5’-ACC TAT GAC TAC CAA AAG CTC ATG TAG AAG C-3’	Arévalo et al. (1994)
		H12763V: 5’-TTC TAT CAC TTG GAT TTG CAC CA-3’	Barlow et al. (2013)
cyt *b*	Yes	L14910: 5’- GAC CTG TGA TMT GAA AAA CCA YCG TTG T-3’	Burbrink et al. (2000)
		LycodryasG3R: 5’-TGG AAT GGR ATT TTR TCG AT-3’	Ruane et al. (2015)
SPTBN1	Yes	SPTBN1F APR-2010: 5’-TTGGTC GAT GCC AGT TGT A-3’	Ruane et al. (2014)
		SPTBN1R APR-2010: 5’-CAG GGT TTG TAA CCT KTC CA-3’	Ruane et al. (2014)

### Phylogenetic analysis

The mtDNA and nuDNA sequences were aligned in CLUSTAL W (Larkin et al. 2007) using the default settings. The two protein-coding mtDNA (ND4 and cyt *b*) loci were examined for the presence of pseudogenes by converting the DNA sequence to amino acids to detect the presence of stop codons. Since all loci on the mtDNA were linked, the two mtDNA loci were combined for the phylogenetic analysis. For ND4 and cyt *b*, 740 bp and 610 bp fragment was respectively sequenced for the 87 *D.l.lutrix* specimens. Sequences were deposited in GenBank (Table 1). The combined mtDNA data set yielded a total of 1350 bp. The DNA substitution models obtained in jModelTest 2.0 (Darriba et al. 2012) are provided in Suppl. material 1. In addition, ND4 and cyt *b* sequences for *D.variegata*, as well as an additional specimen of *D.lutrix*. sp from Kenya were downloaded from GenBank and included in the phylogenetic analysis (Kelly et al. 2008; Vidal et al. 2008). For the SPTBN1, the older samples failed to amplify, hence these were coded as missing during the combined analyses. For the nuDNA SPTBN1, a 760 bp fragment was amplified for 30 *D.l.lutrix* specimens and sequences were deposited in GenBank (accession numbers in Table 1). For the nuclear SPTBN1 locus, allelic heterozygotes were inferred using PHASE (Stephens et al. 2001; Stephens and Scheet 2005). PHASE implements a Bayesian method for reconstructing haplotypes from nuclear sequences that include multiple heterozygous base sites within individuals. To estimate allele frequencies, PHASE was run five times. The run with the best goodness-of-fit to an approximate coalescent model was retained, resulting in two nuclear haplotype sequences of alleles per individual.

The combined mtDNA data set was subjected to a Bayesian inference (BI). The BI analysis was performed in MrBayes v3.2.6 (Ronquist et al. 2012; Huelsenbeck et al. 2016), sampling every 10,000 generations for a total of 10 million generations. Convergence and burn-in were determined using Tracer v1.6 (Rambaut et al. 2014). Nodes were considered well supported when they had a posterior probability (p*P*) of > 0.95. All trees were visualised using TreeGraph 2 (Stöver and Müller 2010). For the combined DNA analysis, a single specimen per locality was used of each of the three loci and the same phylogenetic approaches listed above were followed to reconstruct evolutionary relationships. In addition, we also performed a maximum parsimony (MP) analyses on the combined mtDNA data and the total evidence data sets. MP analyses were executed in PAUP*4 v. beta 10 (Swofford 2002). For the MP analyses, trees were generated using the heuristic search option with tree bisection and reconnection (TBR branch swapping using 100 random taxon additions). Phylogenetic confidence in the nodes recovered from MP was estimated by bootstrapping (Felsenstein 1985) analysing 1000 replicates of the data set. Only bootstrap values >75% were regarded as statistically well supported (Felsenstein 1985).

Both Vidal et al. (2008) and Kelly et al. (2009) demonstrated that the two snake species *Amplorhinusmultimaculatus* and *Ditypophisvivax* are sister to *Duberria*. Hence the former two species were used as outgroups. Mitochondrial DNA sequences for the latter two species were downloaded from GenBank whereas the nuDNA locus was coded absent. Uncorrected sequence divergence values for both the ND4 and the cyt *b* loci were calculated in PAUP*4 version beta 10 (Swofford 2002). We did not combine the two mtDNA to calculate the sequence divergence value since we would not be able to compare this value with other phylogeographic studies since most authors perform this analysis on loci individually.

### Population and demographic analysis

Haplotype networks were constructed for the combined mtDNA data, as well as for the nuclear SPTBN1 using TCS version 1.3 using a 95% connection limit (Clement et al. 2000). An analysis of molecular variance (AMOVA) was conducted in ARLEQUIN v3.5.2.2 (Excoffier and Lischer 2010) using the combined mtDNA data. The preliminary analyses of the combined mtDNA topology revealed the presence of five clades, hence a hierarchical AMOVA was performed; 1) across all sample localities and; 2) for haploclade one detected using the combined mtDNA data set. The remaining four clades were not analysed further in AMOVA since the low sample sizes limited any statistical inferences. Two neutrality test using Fu’s *Fs* (Fu 1997) and Tajima’s D (Tajima 1989) were calculated in ARLEQUIN using 10,000 permutations.

### Divergence time estimations

We used BEAST v1.8.3 (Drummond et al. 2012) on the mitochondrial data set to determine the age of divergent events within *Duberrial.lutrix*. No fossil calibrations points are available for *Duberria*. The genus *Duberria* belongs to the subfamily Pseudoxyrhophiinae which is part of the family Lamprophiidae. The Lamprophiidae is in turn sister to the Elapidae and both being sister to the superfamily Colubroidea. Hence published mutation rates from the superfamily were used in the divergence time estimation (Figueroa et al. 2016; Hsaing et al. 2015 For the two mtDNA loci (ND4 and cyt *b*) a strict substitution rate of 1.34% (SD=0.251) per million years was used (Daza et al. 2009) after having checked that the relaxed log-normal clock’s standard deviation approached zero. The mutation rate for the nuclear marker is unknown, hence this marker was excluded from the divergence time estimation. We ran the analyses under the substitution model as inferred above (TrN+Gamma), unlinked between both mitochondrial genes, and under a coalescent prior with constant population size, as the resulting tree described within species relationships. We ran the Markov chain for 50 million iterations and sampled every 10,000 iteration. Tracer v1.6 (Rambaut et al. 2014) was used to assess chain convergence to ensure minimal autocorrelation between iterations (effective sample size > 2000 for all sampled parameters) and to determine the burn in (10% of samples). TreeAnnotator in BEAST was used to determine a maximum clade credible tree.

## Results

### Combined mtDNA analyses (*ND4* and *cyt* b)

**Figure 2.**
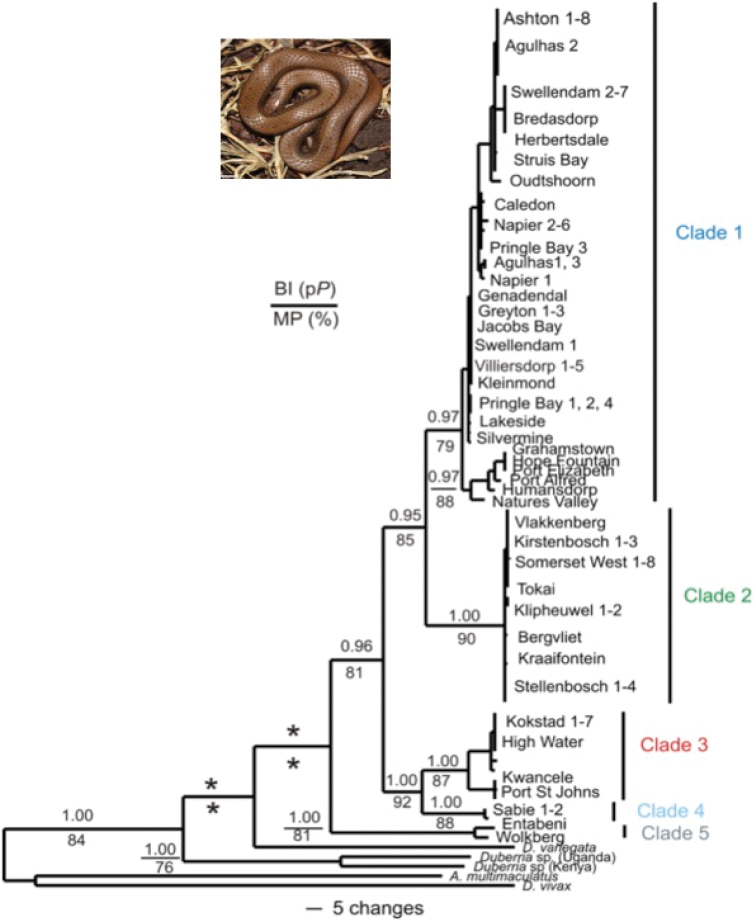
A Bayesian inference topology derived from the combined mtDNA analyses (ND4 + cyt *b*) amongst the South African *Duberrial.lutrix*. The posterior probability value (p*P*) values are presented above each node. Only p*P* values >0.95 are shown. Values below each node are bootstrap values for MP. Only bootstrap values >75% are shown. An asterisk (*) below or above a node indicates the absence of statistical support. The insert shows the typical *Duberrialutrixlutrix*.

The MP and BI analyses retrieved near identical tree topologies, hence only the BI topology is shown and discussed. For MP, of a total of 1350 characters, 278 characters were found to be parsimony informative, and recovered 167 trees with a tree length of 597 steps with a consistency index (CI) of 0.59 and a retention index (RI) of 0.88. The BI topology (Fig. 2) retrieved the two East African specimens (Kenya and Uganda) of *Duberria* as basal, while *D.variegata* appeared sister to a South African clade of *D.l.lutrix*, with low statistical support for the monophyly of the clade. Within *D.l.lutrix*, five geographically discrete, statistically well-supported clades were detected (>75%/>0.95 p*P*). Clade one consisted of specimens that occurred predominantly from above the Hottentots Holland Mountains, Agulhas plain and Overberg, the Cape Peninsula and the south-eastern Cape and adjacent interior, and was sister to clade two. Clade two comprised specimens from below the Hottentots Holland Mountains, including the Cape Peninsula and Boland region. Clade three was comprised of specimens from the Eastern Cape coast and samples from interior of KwaZulu-Natal Province, and was sister to clade four. Clade four comprised two specimens from Mpumalanga (Sabie), while clade five comprised two specimens exclusive to the Limpopo Province (Entabeni and Wolkberg).

### Population genetic analysis using the combined mtDNA

For the ND4 locus the maximum uncorrected sequence divergence between the first and second clades was 1.68%. Between the second and third clade the maximum uncorrected sequence divergence was 3.84%. Between the third and the fourth clades the maximum uncorrected sequence divergence was 5.95%. Finally, the maximum uncorrected sequence divergence between clades four and five was 6.75% (Suppl. material 1: Table S1). For cyt *b* locus the maximum uncorrected sequence divergence between the first and second clades was 0.98%. Between the second and third clade the maximum uncorrected sequence divergence was 2.62%. Between the third and the fourth clades the maximum uncorrected sequence divergence was 3.77%. The maximum uncorrected sequence divergence between clades four and five was 6.01% (Suppl. material 2: Table S2). The ND4 locus revealed slightly higher levels of uncorrected sequence divergence values during the present study in comparison to the cyt *b* locus.

**Figure 3.**
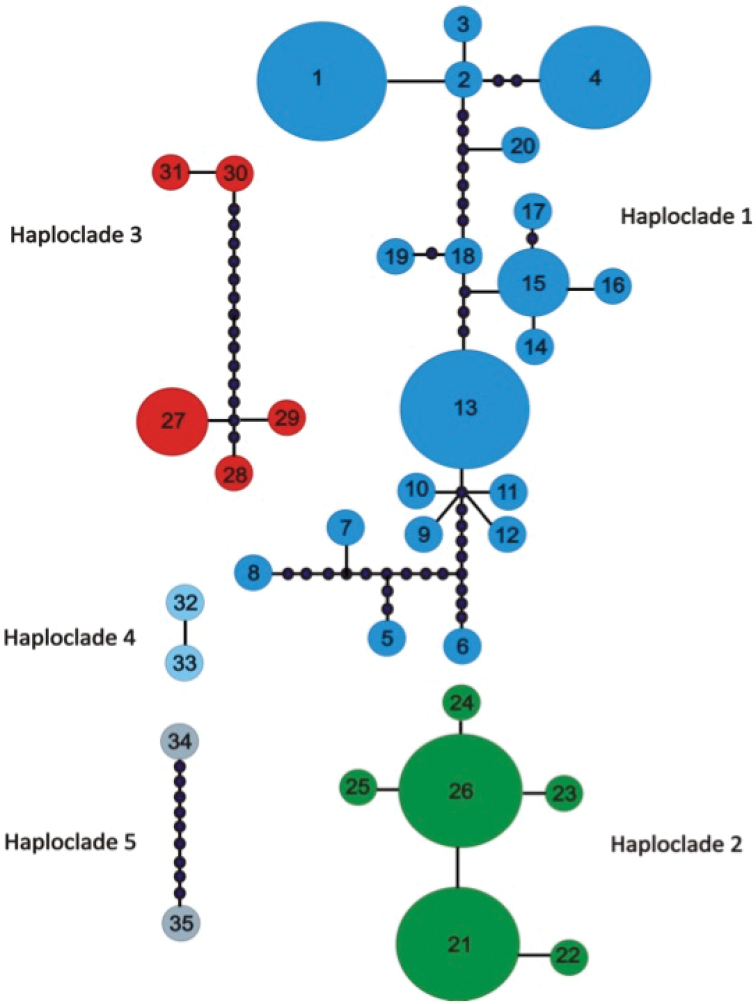
A minimum spanning network derived from combined mtDNA (ND 4 + cyt *b*) analyses demonstrating the five haploclades for *Duberrial.lutrix*. The number inside the boxes correspond to the haplotypes in Table 3. The closed black dots represent unsampled or missing haplotypes.

A total of 35 haplotypes were retrieved for the 87 *Duberrial.lutrix* specimens using the combined mtDNA (Fig. 3). For details of the haplotype distribution consult the Suppl. material 1: Table S3. Five haplogroups were retrieved, revealing a pattern congruent with the combined mtDNA topology (Fig. 2). The AMOVA results among all 38 sample localities revealed that 94.26% (Va = 15.89; df = 37; SS = 1394.37) of the variation occurred among sample sites, while 5.74% (Vb = 0.96; df = 49; SS = 47.44) of the variation occurred within sample sites. These results are indicative of marked genetic differentiation, a result that is corroborated by the marked Φst (0.94) as well as the high *F*_ST_ values among sample localities that were statistically significant for 56 combinations, ranging from 0.05 to 0.99 (results not shown). Haploclade one (Fig. 3) consisted of 20 haplotypes comprising samples from above the Hottentots Holland Mountains, Agulhas plain and the Cape Peninsula separated by ten unsampled / missing mutations from samples from the south-eastern Western Cape, with 76.46% of the variation occurring among sample localities, (Va = 3.07; df = 22, SS = 167.63), 23.58% occurred within sample localities (Vb = 0.94; df = 29; SS = 27.53), with a high Φst (0.74) as well as high *F*_ST_ values among sample localities that were generally statistically significant. The remaining four haploclades, two, three, four and five respectively (corresponding to the identical clades observed in Fig. 2) contained fewer than five haplotypes, hence we did not undertake any further statistical analyses due to the low sample sizes.

Fu’s *Fs* values were positive for Agulhas, Kokstad, Pringle Bay and Swellendam and indicate an excess of intermediate polymorphisms due to recent population bottlenecks or balancing selection, however none of these were statistically significant. Two of the Fu’s *Fs* values were negative for Somerset West and Napier indicating an excess of low frequency polymorphisms consistent with population expansions or positive directional selection. However, only the Somerset West population was statistically significant (*P* < 0.02). The remaining sample localities had a Fu’s *Fs* of zero. For Tajima’s D, five sample localities, Swellendam, Pringle Bay, Napier, Somerset West and Kokstad were negative, while only Napier and Kokstad were statistically significant (*P* < 0.05). The remaining sample localities has a Tajima’s D of zero. Negative Tajima’s D indicates an excess of low frequency polymorphism, population expansion or purifying selection.

### SPTBN1

Ten haplotypes were retrieved for the 30 specimens using the TCS analyses (network not shown). For a list of the sample localities per haplotype consult Suppl. material 2. Three haploclades were retrieved. Haploclade one contained six haplotypes from all the remaining sample localities (from clades 1, 2, 3 and 4; Fig. 2). Haploclade two contained a single haplotype from Oudtshoorn (clade 1; Fig. 2). Haploclade three contained three haplotypes from localities in the KwaZulu-Natal (Kokstad) and the Limpopo provinces (Wolkberg and Entabeni) (from clades five and three respectively; Fig. 2).

### Total evidence phylogeny (*ND4*, cyt *b* + SPTBN 1)

**Figure 4.**
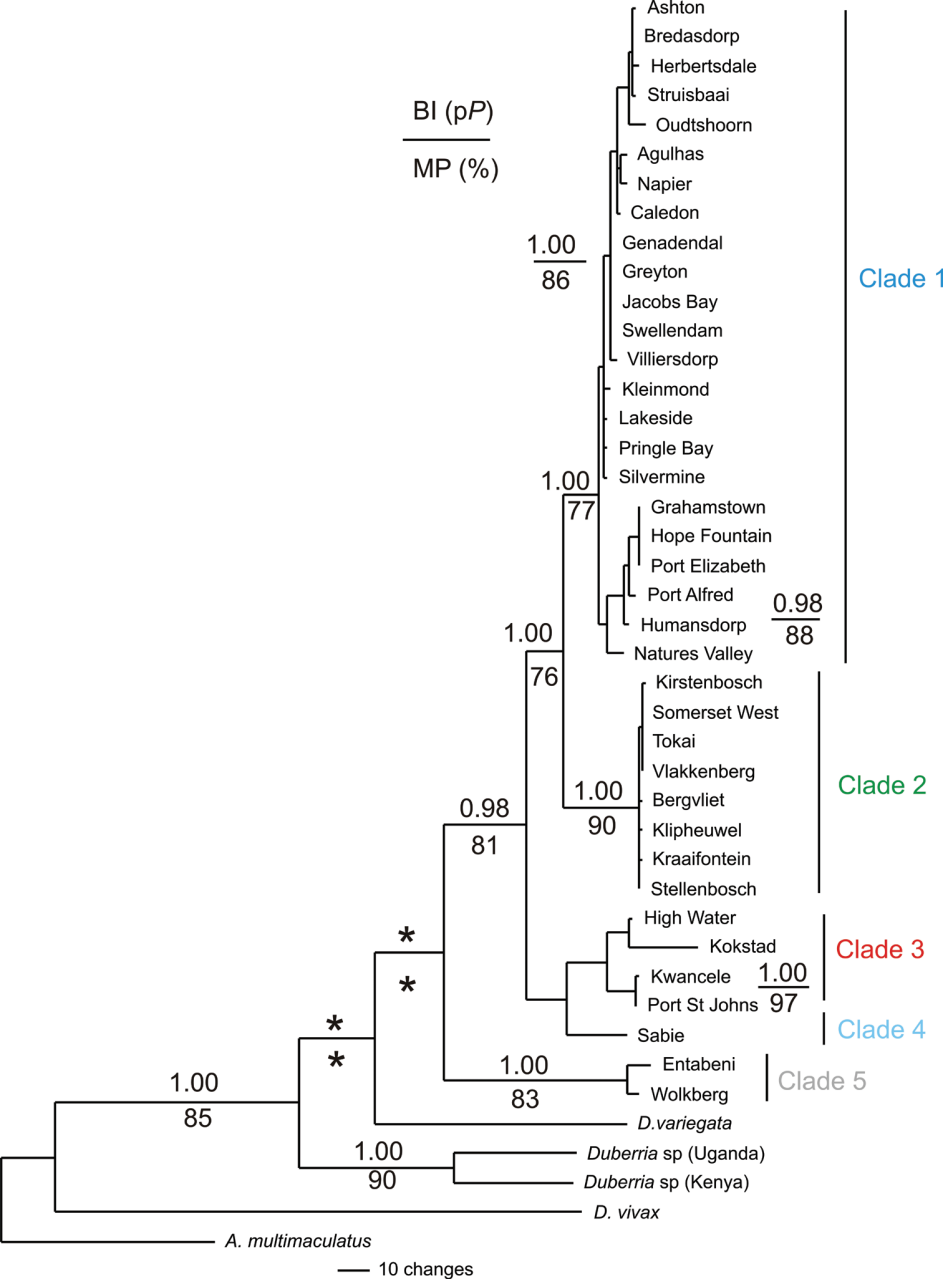
A Bayesian inference topology for the total evidence data sets (ND4 + cyt *b* + SPTBN1) amongst the South African *Duberrial.lutrix* species complex. The posterior probability value (p*P*) values are presented above each node. Only p*P* values >0.95 are shown. Values below each node are bootstrap values for MP. Only bootstrap values >75% are shown. An asterisk (*) below or above a node indicates the absence of statistical support.

The combined mtDNA and nuDNA sequence data yielded a total of 2110 bp. The MP and the BI analyses retrieved highly congruent tree topologies, hence only the BI topology is shown. For the MP analyses, 293 characters were found to be parsimony informative, tree length of 627 steps, 730 trees, with CI = 0.58 and RI = 0.77. The total evidence BI topology (Fig. 4) was congruent with the combined mtDNA topology (Fig. 2). A monophyletic *Duberria* was retrieved with the two East African *Duberria* specimens (*D.l.atriventris*) form a basal split, while *D.variegata* was sister to a clade containing all the *D.l.lutrix* samples from South Africa. These five clades were also evident in the combined mtDNA topology (Fig. 2).

### Divergence time estimate based on total mtDNA data

Clade five diverged from the remaining *D.l.lutrix* clades 3.42 Mya (4.13 Mya to 2.72 Mya; 95% HPD). These divergences dates fall into the Pliocene and Pleistocene epochs. Clade four diverged from clades one, two and three occurred 2.05 Mya (1.34 Mya to 2.95 Mya; 95% HPD). The divergence between clade three from clades one and two occurred 1.23 Mya (0.74 Mya and 1.81 Mya; 95% HPD). Cladogenesis between clades one and two occurred 0.46 Mya (0.27 Mya and 0.69 Mya; 95% HPD) (Suppl. material 3).

## Discussion

### Biogeographic affinities

The phylogeographic results demonstrated the presence of five mtDNA clades across the sampled distribution range of *Duberrial.lutrix* in South Africa, implying that the snake represents a species complex. Furthermore, these five clades were characterised by the absence of shared mtDNA haplotypes and marked sequence divergences values for both the ND4 and cyt *b* loci, suggesting possible genetic isolation and limited dispersal. However, the nuclear DNA sequences data failed to retrieve patterns congruent with the mtDNA data, hence it is not possible to exclusively imply the presence of five possible taxa within the *Duberrial.lutrix* species complex, and requires further taxonomic delineation. In addition, the divergence time estimates suggest that cladogenesis in the *D.l.lutrix* species complex occurred during the Plio/Pleistocene epochs, a period that was characterised by increased aridification throughout South Africa, resulting in the contraction of mesic adapted species. Our results reflect the impact of Plio/Pleistocene climatic driven fragmentation on a snake species resulting in possible cladogenesis. The latter result is in line with what has been reported for the widely distributed puff adder in South Africa (Barlow et al. 2013).

During the late Miocene, climatic profiles changed dramatically, resulting in decreasing levels of precipitation and marked aridification, a trend that was enhanced in the Plio/Pleistocene (Siesser 1980; Marlow et al. 2000; Cowling et al. 2009; Engelbrecht et al. 2013). Furthermore, during the Plio/Pleistocene epochs, coastal regions experienced dramatic marine transgressions that have been estimated to vary between 150–200 meters in certain regions (Partridge and Maud 1987, 2000; Partridge 1997). These events likely resulted in the extinction of low-lying terrestrial taxa characterised by low vagility and habitat specificity and the contraction of animals to high-lying refugial areas along the coastal belt mountains and the adjacent interior. More recently, during periods of glacial maxima in the Holocene, the interior of South Africa is thought to have become more inhospitable to ectotherms, due to low winter temperatures and reduced precipitation levels. These factors possibly caused mesic adapted organisms such as *D.l.lutrix* to seek more favourable habitat along the high-lying mountainous coastal regions of the Cape Fold and Drakensberg Mountains (Barlow et al. 2013; Tolley et al. 2014). During the Last Glacial Maximum coastal areas would have had exposed areas of continental shelf, off the current south-west and western coasts of South Africa, due to the lowering sea levels. This would have provided favourable habitat, as well as, acting as dispersal corridors for many species (Schreiner et al. 2013). During the interglacial period these corridors would have been inaccessible due to the rising of sea levels. Rapid changes in elevation can provide significant biogeographic barriers to the dispersal of ectotherms; this is evident when one compares the geographic topology of clades one and three. Clade one extends from the western coast of the Cape Peninsula above the Hottentots Holland Mountains range until just before the south-eastern Cape coastline whilst clade three occurs below the Hottentots Holland mountain range extending into the Cape Flats. The rapid changes in elevation between the two clades limits the dispersal of *D.l.lutrix*. Similar phylogeographic breaks have been observed in other co-distributed reptile species (Daniels et al. 2007, 2009; Tolley et al. 2009, 2014; Barlow et al. 2013). Climatic fluctuations would have altered environmental conditions during the Plio/Pleistocene allowing for dispersal of *D.l.lutrix* around these mountain ranges due to the changes in sea levels. Further evidence for isolation induced by climatic conditions during the Plio/Pleistocene can be found between clades one and two. Clade one occurs predominately throughout the western half of the Greater Cape Floristic Region (GCFR), namely the Cape Peninsula, above the Hottentots Holland mountain ranges and throughout the Agulhas plains and Klein Karoo, whilst clade two occurs within the eastern half of the GCFR, along the south-eastern Cape coastline and interior. Potentially, the reason for the genetic isolation between the two clades may be due to changes in the climatic conditions across the GCFR. The western and eastern sections of the GCFR are characterised by distinct rainfall regimes with the western half being characterised by a winter rainfall, while the eastern half is characterised by aseasonal and/or summer rainfall regime (Siesser 1980; Cowling et al. 2009; Tolley et al. 2009). It has been hypothesised that the division between some clades of reptiles corresponds to the changes in the rainfall regimes (Tolley et al. 2009, 2014). This is further corroborated by clades two and four which are found in the Eastern Cape, KwaZulu-Natal and Mpumalanga provinces. The Bedford gap is situated between the two clades. However, it is uncertain whether the genetic isolation is due to a combination of the xeric conditions and changes in rainfall patterning or simply due to one of the two variables. As rainfall patterns change and environments become more xeric, the minimum annual temperature of the area decreases, which can potentially limit the dispersal capabilities, as well as, survival capabilities of ectotherms. Furthermore, this would explain why *D.l.lutrix* has not dispersed along the western coastline of South Africa where the average annual precipitation and minimum annual temperatures are lower. This can be further evaluated by observing the effects that the Breede River xeric corridor had on the phylogenetic patterning of *D.l.lutrix*. When examining the topology of the phylogenetic trees (Figs 2, 4) the Breede River xeric corridor did not display any pronounced impact on the phylogeographic patterning. This observation favours the change in rainfall patterning as a possible source for the genetic isolation observed within *D.l.lutrix*. However, climatic fluctuations during interglacial periods would have lessened the impact that this xeric valley had on the dispersal of this species as precipitation levels changed. Finally, the extinction of intermediary haplotypes, possibly due to the climatic fluctuations, in widely distributed species with low dispersal capabilities and gene flow may have resulted in these pronounced phylogenetic gaps. Widespread sampling of *D.l.lutrix* is required to affirm or reject these inferences.

Although the climatic fluctuation would have forced species to retreat into refugia (Barlow et al. 2013; Tolley et al. 2014), evidence for the relictual populations, which are proposed to be restricted to the interior in the Klein Karoo and Free State are not corroborated by our results. The haplotype network for the nuclear marker SPTBN1 showed the Klein Karoo locality to be isolated from the other localities in the western and south-eastern Cape coastline and interior. However, this can be potentially biased; as firstly, the SPTBN1 is a protein coding locus and secondly, the mutation rates for nuclear markers in ectotherms are much slower than the mitochondrial markers, limiting inferences derived from them.

### Cryptic diversity and taxonomy

The combined mitochondrial and nuclear data set retrieved five clades that show evidence for geographically distinct *Duberrialutrixlutrix* lineages. The mtDNA data shows, high levels of uncorrected sequence divergence values. Glaw et al. (2007) reported that within the Malagasy *Geodipsasinfralineata* using the cyt *b* marker uncorrected sequence divergence between the two clades ranged between 4.7–4.8%. Similarly, Ruane et al. (2017) using morphology and DNA sequencing (of the cytochrome oxidase one locus) observed two clades within the widespread Malagasy snake *Mimophismahfalensis*. It is noteworthy, that while the phylogenetic affinities within the Lamprophiidae has recently received attention (Vidal et al. 2008; Portillo et al. 2018), phylogeographic studies remain limited. The latter observation suggests that species diversity among widespread species in the family may have resulted in an underestimation of alpha taxonomic diversity.

These sequence divergence values are similar to values observed in other snake lineages within Colubroidea and the Malagasy Pseudoxyrhophiinae that are considered genetically different (Feldman and Spicer 2002; Gou et al. 2011; Kindler et al. 2013; Ruane et al. 2015). This indicates that the respective clades might potentially be composed of cryptic species; however, we are cautious of the pitfalls of using these divergence estimations as exclusive evidence for species boundaries. Our results partially support the observation by Branch (2002, 2003), that the clade to which specimens from Port St Johns belong may represent a cryptic lineage. However, considering the sparse sampling of *D.l.lutrix* during the present study this might be an underestimation of species diversity. We advocate larger sample sizes per sample locality and a more comprehensive geographic sampling of the species range throughout South Africa coupled with more sensitive nuclear DNA markers, such as microsatellites or single nucleotide polymorphisms (SNP’s) to examine patterns of biparental gene flow. However, it is frequently difficult to obtain large sample sizes for snakes, specifically for phylogeographic studies. Similar observation has been made in other studies on snakes (Martínez-Freiría et al. 2015).

## Supplementary materials

Supplementary material 1 Uncorrected ("p") distance matrix for the ND4 locus for all the *Duberrialutrixlutrix* samples used during the present study Data type: molecular data This dataset is made available under the Open Database License (http://opendatacommons.org/licenses/odbl/1.0/). The Open Database License (ODbL) is a license agreement intended to allow users to freely share, modify, and use this Dataset while maintaining this same freedom for others, provided that the original source and author(s) are credited.Kyle Kulenkampff, Francois Van Zyl, Sebastian Klaus, Savel R. Daniels

Supplementary material 2 Uncorrected “p” distances for the cyt b for the *Duberrialutrixlutrix* sampled during the present study Data type: molecular data This dataset is made available under the Open Database License (http://opendatacommons.org/licenses/odbl/1.0/). The Open Database License (ODbL) is a license agreement intended to allow users to freely share, modify, and use this Dataset while maintaining this same freedom for others, provided that the original source and author(s) are credited.Kyle Kulenkampff, Francois Van Zyl, Sebastian Klaus, Savel R. Daniels

Supplementary material 3 Distribution of the 35 haplotype frequency for the combined mtDNA loci sequence data for the 87 *Duberrialutrixlutrix* specimens Data type: molecular data Explanation note: The haplotype numbers (N) corresponds to the numbers on the minimum spanning network (Fig. 3). This dataset is made available under the Open Database License (http://opendatacommons.org/licenses/odbl/1.0/). The Open Database License (ODbL) is a license agreement intended to allow users to freely share, modify, and use this Dataset while maintaining this same freedom for others, provided that the original source and author(s) are credited.Kyle Kulenkampff, Francois Van Zyl, Sebastian Klaus, Savel R. Daniels
