# Supplementary material for: Molecular evidence for cryptic species in the common slug eating snake Duberrialutrixlutrix (Squamata, Lamprophiidae) from South Africa
Source: Zookeys. 2019 Apr 15;838:133–54. doi: 10.3897/zookeys.838.32022 (PMC6477839; doi:10.3897/zookeys.838.32022)
Supplement: Supplementary material 1 [file zookeys-838-133-s001.docx]

Supplementary table 1. Uncorrected ("p") distance matrix for the ND4 locus for all the *Duberria lutrix lutrix* samples used during the present study

1 2 3 4 5 6 7 8

1 A.mltmclts -

2 D.vivax 0.21818 -

3 Ashton1 0.16907 0.20278 -

4 Ashton2 0.16907 0.20278 0.00000 -

5 Ashton3 0.16907 0.20278 0.00000 0.00000 -

6 Ashton4 0.16907 0.20278 0.00000 0.00000 0.00000 -

7 Ashton5 0.16907 0.20278 0.00000 0.00000 0.00000 0.00000 -

8 Ashton6 0.16907 0.20278 0.00000 0.00000 0.00000 0.00000 0.00000 -

9 Ashton7 0.16907 0.20278 0.00000 0.00000 0.00000 0.00000 0.00000 0.00000

10 Ashton8 0.16907 0.20278 0.00000 0.00000 0.00000 0.00000 0.00000 0.00000

11 Bergvliet1 0.18023 0.20313 0.03649 0.03649 0.03649 0.03649 0.03649 0.03649

12 Caledon1 0.16276 0.20092 0.00541 0.00541 0.00541 0.00541 0.00541 0.00541

13 Entabeni1 0.17664 0.21044 0.06892 0.06892 0.06892 0.06892 0.06892 0.06892

14 Flakkenberg1 0.18171 0.20462 0.03784 0.03784 0.03784 0.03784 0.03784 0.03784

15 Genadendal1 0.16421 0.19945 0.00676 0.00676 0.00676 0.00676 0.00676 0.00676

16 Greyton1 0.16421 0.19945 0.00676 0.00676 0.00676 0.00676 0.00676 0.00676

17 Greyton2 0.16421 0.19945 0.00676 0.00676 0.00676 0.00676 0.00676 0.00676

18 Greyton3 0.16421 0.19945 0.00676 0.00676 0.00676 0.00676 0.00676 0.00676

19 Herbertsdale1 0.17075 0.20446 0.00135 0.00135 0.00135 0.00135 0.00135 0.00135

20 HighWaters1 0.17757 0.19690 0.05270 0.05270 0.05270 0.05270 0.05270 0.05270

21 HopeFountain1 0.17044 0.20131 0.01351 0.01351 0.01351 0.01351 0.01351 0.01351

22 Humansdorp1 0.17025 0.20244 0.01351 0.01351 0.01351 0.01351 0.01351 0.01351

23 Jacobsbaai1 0.16421 0.19945 0.00676 0.00676 0.00676 0.00676 0.00676 0.00676

24 Kirstenbosch1 0.18171 0.20462 0.03784 0.03784 0.03784 0.03784 0.03784 0.03784

25 Kirstenbosch2 0.18171 0.20462 0.03784 0.03784 0.03784 0.03784 0.03784 0.03784

26 Kirstenbosch3 0.18171 0.20462 0.03784 0.03784 0.03784 0.03784 0.03784 0.03784

27 Kokstad1 0.17750 0.19837 0.05135 0.05135 0.05135 0.05135 0.05135 0.05135

28 Kokstad2 0.17750 0.19837 0.05135 0.05135 0.05135 0.05135 0.05135 0.05135

29 Kokstad3 0.17583 0.19822 0.05000 0.05000 0.05000 0.05000 0.05000 0.05000

30 Kokstad4 0.17750 0.19837 0.05135 0.05135 0.05135 0.05135 0.05135 0.05135

31 Kokstad5 0.17750 0.19837 0.05135 0.05135 0.05135 0.05135 0.05135 0.05135

32 Kokstad6 0.18036 0.20161 0.04865 0.04865 0.04865 0.04865 0.04865 0.04865

33 Kokstad7 0.17750 0.19837 0.05135 0.05135 0.05135 0.05135 0.05135 0.05135

34 Kraaifontein1 0.18023 0.20313 0.03649 0.03649 0.03649 0.03649 0.03649 0.03649

35 Kwancele1 0.18183 0.20307 0.05000 0.05000 0.05000 0.05000 0.05000 0.05000

36 LakesideCT1 0.16273 0.19798 0.00811 0.00811 0.00811 0.00811 0.00811 0.00811

37 Napier1 0.16128 0.19945 0.00676 0.00676 0.00676 0.00676 0.00676 0.00676

38 Napier2 0.16276 0.20092 0.00541 0.00541 0.00541 0.00541 0.00541 0.00541

39 Napier3 0.16276 0.20092 0.00541 0.00541 0.00541 0.00541 0.00541 0.00541

40 Napier4 0.16276 0.20092 0.00541 0.00541 0.00541 0.00541 0.00541 0.00541

41 Napier5 0.16424 0.20239 0.00676 0.00676 0.00676 0.00676 0.00676 0.00676

42 Napier6 0.16276 0.20092 0.00541 0.00541 0.00541 0.00541 0.00541 0.00541

Supplementary table 1 continues.

43 NaturesValley1 0.16761 0.19979 0.00946 0.00946 0.00946 0.00946 0.00946 0.00946

44 Oudtshoorn1 0.17075 0.20586 0.00405 0.00405 0.00405 0.00405 0.00405 0.00405

45 PortAlfred1 0.16898 0.20278 0.01216 0.01216 0.01216 0.01216 0.01216 0.01216

46 PortStJohns 0.18036 0.20161 0.04865 0.04865 0.04865 0.04865 0.04865 0.04865

47 PringleBay1 0.16421 0.19945 0.00946 0.00946 0.00946 0.00946 0.00946 0.00946

48 PringleBay2 0.16273 0.19798 0.00811 0.00811 0.00811 0.00811 0.00811 0.00811

49 PringleBay3 0.16276 0.20092 0.00541 0.00541 0.00541 0.00541 0.00541 0.00541

50 PringleBay4 0.16273 0.19798 0.00811 0.00811 0.00811 0.00811 0.00811 0.00811

51 Sabie1 0.17714 0.19977 0.04189 0.04189 0.04189 0.04189 0.04189 0.04189

52 Sabie2 0.17714 0.19977 0.04189 0.04189 0.04189 0.04189 0.04189 0.04189

53 Silvermine 0.16273 0.19798 0.00811 0.00811 0.00811 0.00811 0.00811 0.00811

54 SomersetWest1 0.18171 0.20462 0.03784 0.03784 0.03784 0.03784 0.03784 0.03784

55 SomersetWest2 0.18177 0.20476 0.03919 0.03919 0.03919 0.03919 0.03919 0.03919

56 SomersetWest3 0.18023 0.20313 0.03649 0.03649 0.03649 0.03649 0.03649 0.03649

57 SomersetWest4 0.18023 0.20313 0.03649 0.03649 0.03649 0.03649 0.03649 0.03649

58 SomersetWest5 0.18171 0.20462 0.03784 0.03784 0.03784 0.03784 0.03784 0.03784

59 SomersetWest6 0.18171 0.20462 0.03784 0.03784 0.03784 0.03784 0.03784 0.03784

60 SomersetWest7 0.18023 0.20313 0.03649 0.03649 0.03649 0.03649 0.03649 0.03649

61 Stellenbosch1 0.18023 0.20313 0.03649 0.03649 0.03649 0.03649 0.03649 0.03649

62 Stellenbosch2 0.18023 0.20313 0.03649 0.03649 0.03649 0.03649 0.03649 0.03649

63 Stellenbosch3 0.18023 0.20313 0.03649 0.03649 0.03649 0.03649 0.03649 0.03649

64 Stellenbosch4 0.18023 0.20313 0.03649 0.03649 0.03649 0.03649 0.03649 0.03649

65 Swellendam1 0.16421 0.19945 0.00676 0.00676 0.00676 0.00676 0.00676 0.00676

66 Tokai1 0.18171 0.20462 0.03784 0.03784 0.03784 0.03784 0.03784 0.03784

67 Uganda1 0.19051 0.22237 0.08919 0.08919 0.08919 0.08919 0.08919 0.08919

68 Villiersdorp1 0.16421 0.19945 0.00676 0.00676 0.00676 0.00676 0.00676 0.00676

69 Villiersdorp2 0.16421 0.19945 0.00676 0.00676 0.00676 0.00676 0.00676 0.00676

70 Villiersdorp3 0.16421 0.19945 0.00676 0.00676 0.00676 0.00676 0.00676 0.00676

71 Villiersdorp4 0.16421 0.19945 0.00676 0.00676 0.00676 0.00676 0.00676 0.00676

72 Villiersdorp5 0.16421 0.19945 0.00676 0.00676 0.00676 0.00676 0.00676 0.00676

73 KENYA 0.17779 0.21282 0.10405 0.10405 0.10405 0.10405 0.10405 0.10405

74 D.VARIEGATA 0.19733 0.21973 0.10068 0.10068 0.10068 0.10068 0.10068 0.10068

75 PortElizabeth 0.17121 0.20152 0.01513 0.01513 0.01513 0.01513 0.01513 0.01513

76 Wolkberg 0.17360 0.20742 0.06622 0.06622 0.06622 0.06622 0.06622 0.06622

77 Agulhas1 0.16128 0.19945 0.00676 0.00676 0.00676 0.00676 0.00676 0.00676

78 Agulhas2 0.17227 0.20443 0.00270 0.00270 0.00270 0.00270 0.00270 0.00270

79 Agulhas3 0.16128 0.19945 0.00676 0.00676 0.00676 0.00676 0.00676 0.00676

80 Bredasdorp1 0.16907 0.20278 0.00000 0.00000 0.00000 0.00000 0.00000 0.00000

81 Grahamstown1 0.17044 0.20131 0.01351 0.01351 0.01351 0.01351 0.01351 0.01351

82 Kleinmond1 0.16273 0.19798 0.00811 0.00811 0.00811 0.00811 0.00811 0.00811

83 Klipheuwel1 0.17876 0.20314 0.03784 0.03784 0.03784 0.03784 0.03784 0.03784

84 Klipheuwel2 0.18023 0.20313 0.03649 0.03649 0.03649 0.03649 0.03649 0.03649

85 SomersetWest8 0.17876 0.20314 0.03784 0.03784 0.03784 0.03784 0.03784 0.03784

86 Struisbaai1 0.16907 0.20278 0.00000 0.00000 0.00000 0.00000 0.00000 0.00000

87 Swellendam2 0.17227 0.20443 0.00270 0.00270 0.00270 0.00270 0.00270 0.00270

88 Swellendam3 0.17227 0.20443 0.00270 0.00270 0.00270 0.00270 0.00270 0.00270

89 Swellendam4 0.16907 0.20278 0.00000 0.00000 0.00000 0.00000 0.00000 0.00000

90 Swellendam5 0.17227 0.20443 0.00270 0.00270 0.00270 0.00270 0.00270 0.00270

91 Swellendam6 0.17227 0.20443 0.00270 0.00270 0.00270 0.00270 0.00270 0.00270

92 Swellendam7 0.17227 0.20443 0.00270 0.00270 0.00270 0.00270 0.00270 0.00270

Supplementary table 1 continues.

9 10 11 12 13 14 15 16

9 Ashton7 -

10 Ashton8 0.00000 -

11 Bergvliet1 0.03649 0.03649 -

12 Caledon1 0.00541 0.00541 0.03378 -

13 Entabeni1 0.06892 0.06892 0.06622 0.06351 -

14 Flakkenberg1 0.03784 0.03784 0.00135 0.03514 0.06757 -

15 Genadendal1 0.00676 0.00676 0.03243 0.00135 0.06216 0.03378 -

16 Greyton1 0.00676 0.00676 0.03243 0.00135 0.06216 0.03378 0.00000 -

17 Greyton2 0.00676 0.00676 0.03243 0.00135 0.06216 0.03378 0.00000 0.00000

18 Greyton3 0.00676 0.00676 0.03243 0.00135 0.06216 0.03378 0.00000 0.00000

19 Herbertsdale1 0.00135 0.00135 0.03784 0.00676 0.07027 0.03919 0.00811 0.00811

20 HighWaters1 0.05270 0.05270 0.05811 0.04730 0.06757 0.05946 0.04595 0.04595

21 HopeFountain1 0.01351 0.01351 0.03514 0.01081 0.06892 0.03649 0.00946 0.00946

22 Humansdorp1 0.01351 0.01351 0.03514 0.01081 0.07162 0.03649 0.00946 0.00946

23 Jacobsbaai1 0.00676 0.00676 0.03243 0.00135 0.06216 0.03378 0.00000 0.00000

24 Kirstenbosch1 0.03784 0.03784 0.00135 0.03514 0.06757 0.00000 0.03378 0.03378

25 Kirstenbosch2 0.03784 0.03784 0.00135 0.03514 0.06757 0.00000 0.03378 0.03378

26 Kirstenbosch3 0.03784 0.03784 0.00135 0.03514 0.06757 0.00000 0.03378 0.03378

27 Kokstad1 0.05135 0.05135 0.05676 0.04595 0.06622 0.05811 0.04459 0.04459

28 Kokstad2 0.05135 0.05135 0.05676 0.04595 0.06622 0.05811 0.04459 0.04459

29 Kokstad3 0.05000 0.05000 0.05541 0.04459 0.06622 0.05676 0.04324 0.04324

30 Kokstad4 0.05135 0.05135 0.05676 0.04595 0.06622 0.05811 0.04459 0.04459

31 Kokstad5 0.05135 0.05135 0.05676 0.04595 0.06622 0.05811 0.04459 0.04459

32 Kokstad6 0.04865 0.04865 0.05676 0.04324 0.06486 0.05811 0.04189 0.04189

33 Kokstad7 0.05135 0.05135 0.05676 0.04595 0.06622 0.05811 0.04459 0.04459

34 Kraaifontein1 0.03649 0.03649 0.00000 0.03378 0.06622 0.00135 0.03243 0.03243

35 Kwancele1 0.05000 0.05000 0.05811 0.04459 0.06622 0.05946 0.04324 0.04324

36 LakesideCT1 0.00811 0.00811 0.03108 0.00270 0.06081 0.03243 0.00135 0.00135

37 Napier1 0.00676 0.00676 0.03514 0.00135 0.06486 0.03649 0.00270 0.00270

38 Napier2 0.00541 0.00541 0.03378 0.00000 0.06351 0.03514 0.00135 0.00135

39 Napier3 0.00541 0.00541 0.03378 0.00000 0.06351 0.03514 0.00135 0.00135

40 Napier4 0.00541 0.00541 0.03378 0.00000 0.06351 0.03514 0.00135 0.00135

41 Napier5 0.00676 0.00676 0.03514 0.00135 0.06216 0.03649 0.00270 0.00270

42 Napier6 0.00541 0.00541 0.03378 0.00000 0.06351 0.03514 0.00135 0.00135

43 NaturesValley1 0.00946 0.00946 0.03108 0.00676 0.06486 0.03243 0.00541 0.00541

44 Oudtshoorn1 0.00405 0.00405 0.03784 0.00676 0.07027 0.03919 0.00811 0.00811

45 PortAlfred1 0.01216 0.01216 0.03378 0.00946 0.07027 0.03514 0.00811 0.00811

46 PortStJohns 0.04865 0.04865 0.05676 0.04324 0.06486 0.05811 0.04189 0.04189

47 PringleBay1 0.00946 0.00946 0.03243 0.00405 0.06216 0.03378 0.00270 0.00270

48 PringleBay2 0.00811 0.00811 0.03108 0.00270 0.06081 0.03243 0.00135 0.00135

49 PringleBay3 0.00541 0.00541 0.03378 0.00000 0.06351 0.03514 0.00135 0.00135

50 PringleBay4 0.00811 0.00811 0.03108 0.00270 0.06081 0.03243 0.00135 0.00135

51 Sabie1 0.04189 0.04189 0.05270 0.03649 0.06757 0.05405 0.03514 0.03514

52 Sabie2 0.04189 0.04189 0.05270 0.03649 0.06757 0.05405 0.03514 0.03514

53 Silvermine 0.00811 0.00811 0.03108 0.00270 0.06081 0.03243 0.00135 0.00135

54 SomersetWest1 0.03784 0.03784 0.00135 0.03514 0.06757 0.00000 0.03378 0.03378

55 SomersetWest2 0.03919 0.03919 0.00270 0.03649 0.06757 0.00135 0.03514 0.03514

56 SomersetWest3 0.03649 0.03649 0.00000 0.03378 0.06622 0.00135 0.03243 0.03243

57 SomersetWest4 0.03649 0.03649 0.00000 0.03378 0.06622 0.00135 0.03243 0.03243

58 SomersetWest5 0.03784 0.03784 0.00135 0.03514 0.06757 0.00000 0.03378 0.03378

59 SomersetWest6 0.03784 0.03784 0.00135 0.03514 0.06757 0.00000 0.03378 0.03378

60 SomersetWest7 0.03649 0.03649 0.00000 0.03378 0.06622 0.00135 0.03243 0.03243

61 Stellenbosch1 0.03649 0.03649 0.00000 0.03378 0.06622 0.00135 0.03243 0.03243

62 Stellenbosch2 0.03649 0.03649 0.00000 0.03378 0.06622 0.00135 0.03243 0.03243

63 Stellenbosch3 0.03649 0.03649 0.00000 0.03378 0.06622 0.00135 0.03243 0.03243

64 Stellenbosch4 0.03649 0.03649 0.00000 0.03378 0.06622 0.00135 0.03243 0.03243

65 Swellendam1 0.00676 0.00676 0.03243 0.00135 0.06216 0.03378 0.00000 0.00000

66 Tokai1 0.03784 0.03784 0.00135 0.03514 0.06757 0.00000 0.03378 0.03378

67 Uganda1 0.08919 0.08919 0.08649 0.08919 0.10541 0.08649 0.08784 0.08784

68 Villiersdorp1 0.00676 0.00676 0.03243 0.00135 0.06216 0.03378 0.00000 0.00000

69 Villiersdorp2 0.00676 0.00676 0.03243 0.00135 0.06216 0.03378 0.00000 0.00000

70 Villiersdorp3 0.00676 0.00676 0.03243 0.00135 0.06216 0.03378 0.00000 0.00000

71 Villiersdorp4 0.00676 0.00676 0.03243 0.00135 0.06216 0.03378 0.00000 0.00000

72 Villiersdorp5 0.00676 0.00676 0.03243 0.00135 0.06216 0.03378 0.00000 0.00000

73 KENYA 0.10405 0.10405 0.10541 0.10135 0.10405 0.10676 0.10000 0.10000

74 D.VARIEGATA 0.10068 0.10068 0.09137 0.10052 0.10866 0.09131 0.09900 0.09900

75 PortElizabeth 0.01513 0.01513 0.03693 0.01203 0.07264 0.03842 0.01053 0.01053

76 Wolkberg 0.06622 0.06622 0.06622 0.06081 0.00270 0.06757 0.05946 0.05946

77 Agulhas1 0.00676 0.00676 0.03514 0.00135 0.06486 0.03649 0.00270 0.00270

78 Agulhas2 0.00270 0.00270 0.03919 0.00811 0.07162 0.04054 0.00946 0.00946

79 Agulhas3 0.00676 0.00676 0.03514 0.00135 0.06486 0.03649 0.00270 0.00270

80 Bredasdorp1 0.00000 0.00000 0.03649 0.00541 0.06892 0.03784 0.00676 0.00676

81 Grahamstown1 0.01351 0.01351 0.03514 0.01081 0.06892 0.03649 0.00946 0.00946

82 Kleinmond1 0.00811 0.00811 0.03108 0.00270 0.06081 0.03243 0.00135 0.00135

83 Klipheuwel1 0.03784 0.03784 0.00135 0.03514 0.06757 0.00270 0.03378 0.03378

84 Klipheuwel2 0.03649 0.03649 0.00000 0.03378 0.06622 0.00135 0.03243 0.03243

85 SomersetWest8 0.03784 0.03784 0.00135 0.03514 0.06757 0.00270 0.03378 0.03378

86 Struisbaai1 0.00000 0.00000 0.03649 0.00541 0.06892 0.03784 0.00676 0.00676

87 Swellendam2 0.00270 0.00270 0.03919 0.00811 0.07162 0.04054 0.00946 0.00946

88 Swellendam3 0.00270 0.00270 0.03919 0.00811 0.07162 0.04054 0.00946 0.00946

89 Swellendam4 0.00000 0.00000 0.03649 0.00541 0.06892 0.03784 0.00676 0.00676

90 Swellendam5 0.00270 0.00270 0.03919 0.00811 0.07162 0.04054 0.00946 0.00946

91 Swellendam6 0.00270 0.00270 0.03919 0.00811 0.07162 0.04054 0.00946 0.00946

92 Swellendam7 0.00270 0.00270 0.03919 0.00811 0.07162 0.04054 0.00946 0.00946

Supplementary table 1 continues.

17 18 19 20 21 22 23 24

17 Greyton2 -

18 Greyton3 0.00000 -

19 Herbertsdale1 0.00811 0.00811 -

20 HighWaters1 0.04595 0.04595 0.05405 -

21 HopeFountain1 0.00946 0.00946 0.01486 0.05270 -

22 Humansdorp1 0.00946 0.00946 0.01486 0.05541 0.00541 -

23 Jacobsbaai1 0.00000 0.00000 0.00811 0.04595 0.00946 0.00946 -

24 Kirstenbosch1 0.03378 0.03378 0.03919 0.05946 0.03649 0.03649 0.03378 -

25 Kirstenbosch2 0.03378 0.03378 0.03919 0.05946 0.03649 0.03649 0.03378 0.00000

26 Kirstenbosch3 0.03378 0.03378 0.03919 0.05946 0.03649 0.03649 0.03378 0.00000

27 Kokstad1 0.04459 0.04459 0.05270 0.00135 0.05135 0.05405 0.04459 0.05811

28 Kokstad2 0.04459 0.04459 0.05270 0.00135 0.05135 0.05405 0.04459 0.05811

29 Kokstad3 0.04324 0.04324 0.05135 0.00270 0.05000 0.05270 0.04324 0.05676

30 Kokstad4 0.04459 0.04459 0.05270 0.00135 0.05135 0.05405 0.04459 0.05811

31 Kokstad5 0.04459 0.04459 0.05270 0.00135 0.05135 0.05405 0.04459 0.05811

32 Kokstad6 0.04189 0.04189 0.05000 0.01216 0.04865 0.05135 0.04189 0.05811

33 Kokstad7 0.04459 0.04459 0.05270 0.00135 0.05135 0.05405 0.04459 0.05811

34 Kraaifontein1 0.03243 0.03243 0.03784 0.05811 0.03514 0.03514 0.03243 0.00135

35 Kwancele1 0.04324 0.04324 0.05135 0.01351 0.05000 0.05270 0.04324 0.05946

36 LakesideCT1 0.00135 0.00135 0.00946 0.04459 0.01081 0.01081 0.00135 0.03243

37 Napier1 0.00270 0.00270 0.00811 0.04865 0.01216 0.01216 0.00270 0.03649

38 Napier2 0.00135 0.00135 0.00676 0.04730 0.01081 0.01081 0.00135 0.03514

39 Napier3 0.00135 0.00135 0.00676 0.04730 0.01081 0.01081 0.00135 0.03514

40 Napier4 0.00135 0.00135 0.00676 0.04730 0.01081 0.01081 0.00135 0.03514

41 Napier5 0.00270 0.00270 0.00811 0.04865 0.01216 0.01216 0.00270 0.03649

42 Napier6 0.00135 0.00135 0.00676 0.04730 0.01081 0.01081 0.00135 0.03514

43 NaturesValley1 0.00541 0.00541 0.01081 0.04865 0.00946 0.00946 0.00541 0.03243

44 Oudtshoorn1 0.00811 0.00811 0.00541 0.05135 0.01486 0.01486 0.00811 0.03919

45 PortAlfred1 0.00811 0.00811 0.01351 0.05405 0.00135 0.00405 0.00811 0.03514

46 PortStJohns 0.04189 0.04189 0.05000 0.01216 0.04865 0.05135 0.04189 0.05811

47 PringleBay1 0.00270 0.00270 0.01081 0.04595 0.01216 0.01216 0.00270 0.03378

48 PringleBay2 0.00135 0.00135 0.00946 0.04459 0.01081 0.01081 0.00135 0.03243

49 PringleBay3 0.00135 0.00135 0.00676 0.04730 0.01081 0.01081 0.00135 0.03514

50 PringleBay4 0.00135 0.00135 0.00946 0.04459 0.01081 0.01081 0.00135 0.03243

51 Sabie1 0.03514 0.03514 0.04324 0.03378 0.04189 0.04459 0.03514 0.05405

52 Sabie2 0.03514 0.03514 0.04324 0.03378 0.04189 0.04459 0.03514 0.05405

53 Silvermine 0.00135 0.00135 0.00946 0.04459 0.01081 0.01081 0.00135 0.03243

54 SomersetWest1 0.03378 0.03378 0.03919 0.05946 0.03649 0.03649 0.03378 0.00000

55 SomersetWest2 0.03514 0.03514 0.04054 0.06081 0.03784 0.03784 0.03514 0.00135

56 SomersetWest3 0.03243 0.03243 0.03784 0.05811 0.03514 0.03514 0.03243 0.00135

57 SomersetWest4 0.03243 0.03243 0.03784 0.05811 0.03514 0.03514 0.03243 0.00135

58 SomersetWest5 0.03378 0.03378 0.03919 0.05946 0.03649 0.03649 0.03378 0.00000

59 SomersetWest6 0.03378 0.03378 0.03919 0.05946 0.03649 0.03649 0.03378 0.00000

60 SomersetWest7 0.03243 0.03243 0.03784 0.05811 0.03514 0.03514 0.03243 0.00135

61 Stellenbosch1 0.03243 0.03243 0.03784 0.05811 0.03514 0.03514 0.03243 0.00135

62 Stellenbosch2 0.03243 0.03243 0.03784 0.05811 0.03514 0.03514 0.03243 0.00135

63 Stellenbosch3 0.03243 0.03243 0.03784 0.05811 0.03514 0.03514 0.03243 0.00135

64 Stellenbosch4 0.03243 0.03243 0.03784 0.05811 0.03514 0.03514 0.03243 0.00135

65 Swellendam1 0.00000 0.00000 0.00811 0.04595 0.00946 0.00946 0.00000 0.03378

66 Tokai1 0.03378 0.03378 0.03919 0.05946 0.03649 0.03649 0.03378 0.00000

67 Uganda1 0.08784 0.08784 0.08784 0.09459 0.09189 0.09189 0.08784 0.08649

68 Villiersdorp1 0.00000 0.00000 0.00811 0.04595 0.00946 0.00946 0.00000 0.03378

69 Villiersdorp2 0.00000 0.00000 0.00811 0.04595 0.00946 0.00946 0.00000 0.03378

70 Villiersdorp3 0.00000 0.00000 0.00811 0.04595 0.00946 0.00946 0.00000 0.03378

71 Villiersdorp4 0.00000 0.00000 0.00811 0.04595 0.00946 0.00946 0.00000 0.03378

72 Villiersdorp5 0.00000 0.00000 0.00811 0.04595 0.00946 0.00946 0.00000 0.03378

73 KENYA 0.10000 0.10000 0.10541 0.10135 0.10405 0.10676 0.10000 0.10676

74 D.VARIEGATA 0.09900 0.09900 0.09907 0.11474 0.09910 0.09740 0.09900 0.09131

75 PortElizabeth 0.01053 0.01053 0.01681 0.05512 0.00000 0.00452 0.01053 0.03842

76 Wolkberg 0.05946 0.05946 0.06757 0.06486 0.06622 0.06892 0.05946 0.06757

77 Agulhas1 0.00270 0.00270 0.00811 0.04865 0.01216 0.01216 0.00270 0.03649

78 Agulhas2 0.00946 0.00946 0.00405 0.05541 0.01622 0.01622 0.00946 0.04054

79 Agulhas3 0.00270 0.00270 0.00811 0.04865 0.01216 0.01216 0.00270 0.03649

80 Bredasdorp1 0.00676 0.00676 0.00135 0.05270 0.01351 0.01351 0.00676 0.03784

81 Grahamstown1 0.00946 0.00946 0.01486 0.05270 0.00000 0.00541 0.00946 0.03649

82 Kleinmond1 0.00135 0.00135 0.00946 0.04459 0.01081 0.01081 0.00135 0.03243

83 Klipheuwel1 0.03378 0.03378 0.03919 0.05946 0.03649 0.03649 0.03378 0.00270

84 Klipheuwel2 0.03243 0.03243 0.03784 0.05811 0.03514 0.03514 0.03243 0.00135

85 SomersetWest8 0.03378 0.03378 0.03919 0.05946 0.03649 0.03649 0.03378 0.00270

86 Struisbaai1 0.00676 0.00676 0.00135 0.05270 0.01351 0.01351 0.00676 0.03784

87 Swellendam2 0.00946 0.00946 0.00405 0.05541 0.01622 0.01622 0.00946 0.04054

88 Swellendam3 0.00946 0.00946 0.00405 0.05541 0.01622 0.01622 0.00946 0.04054

89 Swellendam4 0.00676 0.00676 0.00135 0.05270 0.01351 0.01351 0.00676 0.03784

90 Swellendam5 0.00946 0.00946 0.00405 0.05541 0.01622 0.01622 0.00946 0.04054

91 Swellendam6 0.00946 0.00946 0.00405 0.05541 0.01622 0.01622 0.00946 0.04054

92 Swellendam7 0.00946 0.00946 0.00405 0.05541 0.01622 0.01622 0.00946 0.04054

Supplementary table 1 continues.

25 26 27 28 29 30 31 32

25 Kirstenbosch2 -

26 Kirstenbosch3 0.00000 -

27 Kokstad1 0.05811 0.05811 -

28 Kokstad2 0.05811 0.05811 0.00000 -

29 Kokstad3 0.05676 0.05676 0.00135 0.00135 -

30 Kokstad4 0.05811 0.05811 0.00000 0.00000 0.00135 -

31 Kokstad5 0.05811 0.05811 0.00000 0.00000 0.00135 0.00000 -

32 Kokstad6 0.05811 0.05811 0.01081 0.01081 0.01216 0.01081 0.01081 -

33 Kokstad7 0.05811 0.05811 0.00000 0.00000 0.00135 0.00000 0.00000 0.01081

34 Kraaifontein1 0.00135 0.00135 0.05676 0.05676 0.05541 0.05676 0.05676 0.05676

35 Kwancele1 0.05946 0.05946 0.01216 0.01216 0.01351 0.01216 0.01216 0.00135

36 LakesideCT1 0.03243 0.03243 0.04324 0.04324 0.04189 0.04324 0.04324 0.04054

37 Napier1 0.03649 0.03649 0.04730 0.04730 0.04595 0.04730 0.04730 0.04459

38 Napier2 0.03514 0.03514 0.04595 0.04595 0.04459 0.04595 0.04595 0.04324

39 Napier3 0.03514 0.03514 0.04595 0.04595 0.04459 0.04595 0.04595 0.04324

40 Napier4 0.03514 0.03514 0.04595 0.04595 0.04459 0.04595 0.04595 0.04324

41 Napier5 0.03649 0.03649 0.04730 0.04730 0.04595 0.04730 0.04730 0.04459

42 Napier6 0.03514 0.03514 0.04595 0.04595 0.04459 0.04595 0.04595 0.04324

43 NaturesValley1 0.03243 0.03243 0.04730 0.04730 0.04595 0.04730 0.04730 0.04459

44 Oudtshoorn1 0.03919 0.03919 0.05000 0.05000 0.04865 0.05000 0.05000 0.04865

45 PortAlfred1 0.03514 0.03514 0.05270 0.05270 0.05135 0.05270 0.05270 0.05000

46 PortStJohns 0.05811 0.05811 0.01081 0.01081 0.01216 0.01081 0.01081 0.00000

47 PringleBay1 0.03378 0.03378 0.04459 0.04459 0.04324 0.04459 0.04459 0.04189

48 PringleBay2 0.03243 0.03243 0.04324 0.04324 0.04189 0.04324 0.04324 0.04054

49 PringleBay3 0.03514 0.03514 0.04595 0.04595 0.04459 0.04595 0.04595 0.04324

50 PringleBay4 0.03243 0.03243 0.04324 0.04324 0.04189 0.04324 0.04324 0.04054

51 Sabie1 0.05405 0.05405 0.03243 0.03243 0.03108 0.03243 0.03243 0.02568

52 Sabie2 0.05405 0.05405 0.03243 0.03243 0.03108 0.03243 0.03243 0.02568

53 Silvermine 0.03243 0.03243 0.04324 0.04324 0.04189 0.04324 0.04324 0.04054

54 SomersetWest1 0.00000 0.00000 0.05811 0.05811 0.05676 0.05811 0.05811 0.05811

55 SomersetWest2 0.00135 0.00135 0.05946 0.05946 0.05811 0.05946 0.05946 0.05946

56 SomersetWest3 0.00135 0.00135 0.05676 0.05676 0.05541 0.05676 0.05676 0.05676

57 SomersetWest4 0.00135 0.00135 0.05676 0.05676 0.05541 0.05676 0.05676 0.05676

58 SomersetWest5 0.00000 0.00000 0.05811 0.05811 0.05676 0.05811 0.05811 0.05811

59 SomersetWest6 0.00000 0.00000 0.05811 0.05811 0.05676 0.05811 0.05811 0.05811

60 SomersetWest7 0.00135 0.00135 0.05676 0.05676 0.05541 0.05676 0.05676 0.05676

61 Stellenbosch1 0.00135 0.00135 0.05676 0.05676 0.05541 0.05676 0.05676 0.05676

62 Stellenbosch2 0.00135 0.00135 0.05676 0.05676 0.05541 0.05676 0.05676 0.05676

63 Stellenbosch3 0.00135 0.00135 0.05676 0.05676 0.05541 0.05676 0.05676 0.05676

64 Stellenbosch4 0.00135 0.00135 0.05676 0.05676 0.05541 0.05676 0.05676 0.05676

65 Swellendam1 0.03378 0.03378 0.04459 0.04459 0.04324 0.04459 0.04459 0.04189

66 Tokai1 0.00000 0.00000 0.05811 0.05811 0.05676 0.05811 0.05811 0.05811

67 Uganda1 0.08649 0.08649 0.09459 0.09459 0.09595 0.09459 0.09459 0.09459

68 Villiersdorp1 0.03378 0.03378 0.04459 0.04459 0.04324 0.04459 0.04459 0.04189

69 Villiersdorp2 0.03378 0.03378 0.04459 0.04459 0.04324 0.04459 0.04459 0.04189

70 Villiersdorp3 0.03378 0.03378 0.04459 0.04459 0.04324 0.04459 0.04459 0.04189

71 Villiersdorp4 0.03378 0.03378 0.04459 0.04459 0.04324 0.04459 0.04459 0.04189

72 Villiersdorp5 0.03378 0.03378 0.04459 0.04459 0.04324 0.04459 0.04459 0.04189

73 KENYA 0.10676 0.10676 0.10270 0.10270 0.10135 0.10270 0.10270 0.10270

74 D.VARIEGATA 0.09131 0.09131 0.11317 0.11317 0.11141 0.11317 0.11317 0.11617

75 PortElizabeth 0.03842 0.03842 0.05359 0.05359 0.05191 0.05359 0.05359 0.05205

76 Wolkberg 0.06757 0.06757 0.06351 0.06351 0.06351 0.06351 0.06351 0.06216

77 Agulhas1 0.03649 0.03649 0.04730 0.04730 0.04595 0.04730 0.04730 0.04459

78 Agulhas2 0.04054 0.04054 0.05405 0.05405 0.05270 0.05405 0.05405 0.05135

79 Agulhas3 0.03649 0.03649 0.04730 0.04730 0.04595 0.04730 0.04730 0.04459

80 Bredasdorp1 0.03784 0.03784 0.05135 0.05135 0.05000 0.05135 0.05135 0.04865

81 Grahamstown1 0.03649 0.03649 0.05135 0.05135 0.05000 0.05135 0.05135 0.04865

82 Kleinmond1 0.03243 0.03243 0.04324 0.04324 0.04189 0.04324 0.04324 0.04054

83 Klipheuwel1 0.00270 0.00270 0.05811 0.05811 0.05676 0.05811 0.05811 0.05811

84 Klipheuwel2 0.00135 0.00135 0.05676 0.05676 0.05541 0.05676 0.05676 0.05676

85 SomersetWest8 0.00270 0.00270 0.05811 0.05811 0.05676 0.05811 0.05811 0.05811

86 Struisbaai1 0.03784 0.03784 0.05135 0.05135 0.05000 0.05135 0.05135 0.04865

87 Swellendam2 0.04054 0.04054 0.05405 0.05405 0.05270 0.05405 0.05405 0.05135

88 Swellendam3 0.04054 0.04054 0.05405 0.05405 0.05270 0.05405 0.05405 0.05135

89 Swellendam4 0.03784 0.03784 0.05135 0.05135 0.05000 0.05135 0.05135 0.04865

90 Swellendam5 0.04054 0.04054 0.05405 0.05405 0.05270 0.05405 0.05405 0.05135

91 Swellendam6 0.04054 0.04054 0.05405 0.05405 0.05270 0.05405 0.05405 0.05135

92 Swellendam7 0.04054 0.04054 0.05405 0.05405 0.05270 0.05405 0.05405 0.05135

Supplementary table 1 continues.

33 34 35 36 37 38 39 40

33 Kokstad7 -

34 Kraaifontein1 0.05676 -

35 Kwancele1 0.01216 0.05811 -

36 LakesideCT1 0.04324 0.03108 0.04189 -

37 Napier1 0.04730 0.03514 0.04595 0.00405 -

38 Napier2 0.04595 0.03378 0.04459 0.00270 0.00135 -

39 Napier3 0.04595 0.03378 0.04459 0.00270 0.00135 0.00000 -

40 Napier4 0.04595 0.03378 0.04459 0.00270 0.00135 0.00000 0.00000 -

41 Napier5 0.04730 0.03514 0.04595 0.00405 0.00270 0.00135 0.00135 0.00135

42 Napier6 0.04595 0.03378 0.04459 0.00270 0.00135 0.00000 0.00000 0.00000

43 NaturesValley1 0.04730 0.03108 0.04595 0.00405 0.00811 0.00676 0.00676 0.00676

44 Oudtshoorn1 0.05000 0.03784 0.05000 0.00946 0.00811 0.00676 0.00676 0.00676

45 PortAlfred1 0.05270 0.03378 0.05135 0.00946 0.01081 0.00946 0.00946 0.00946

46 PortStJohns 0.01081 0.05676 0.00135 0.04054 0.04459 0.04324 0.04324 0.04324

47 PringleBay1 0.04459 0.03243 0.04324 0.00135 0.00541 0.00405 0.00405 0.00405

48 PringleBay2 0.04324 0.03108 0.04189 0.00000 0.00405 0.00270 0.00270 0.00270

49 PringleBay3 0.04595 0.03378 0.04459 0.00270 0.00135 0.00000 0.00000 0.00000

50 PringleBay4 0.04324 0.03108 0.04189 0.00000 0.00405 0.00270 0.00270 0.00270

51 Sabie1 0.03243 0.05270 0.02703 0.03378 0.03784 0.03649 0.03649 0.03649

52 Sabie2 0.03243 0.05270 0.02703 0.03378 0.03784 0.03649 0.03649 0.03649

53 Silvermine 0.04324 0.03108 0.04189 0.00000 0.00405 0.00270 0.00270 0.00270

54 SomersetWest1 0.05811 0.00135 0.05946 0.03243 0.03649 0.03514 0.03514 0.03514

55 SomersetWest2 0.05946 0.00270 0.06081 0.03378 0.03784 0.03649 0.03649 0.03649

56 SomersetWest3 0.05676 0.00000 0.05811 0.03108 0.03514 0.03378 0.03378 0.03378

57 SomersetWest4 0.05676 0.00000 0.05811 0.03108 0.03514 0.03378 0.03378 0.03378

58 SomersetWest5 0.05811 0.00135 0.05946 0.03243 0.03649 0.03514 0.03514 0.03514

59 SomersetWest6 0.05811 0.00135 0.05946 0.03243 0.03649 0.03514 0.03514 0.03514

60 SomersetWest7 0.05676 0.00000 0.05811 0.03108 0.03514 0.03378 0.03378 0.03378

61 Stellenbosch1 0.05676 0.00000 0.05811 0.03108 0.03514 0.03378 0.03378 0.03378

62 Stellenbosch2 0.05676 0.00000 0.05811 0.03108 0.03514 0.03378 0.03378 0.03378

63 Stellenbosch3 0.05676 0.00000 0.05811 0.03108 0.03514 0.03378 0.03378 0.03378

64 Stellenbosch4 0.05676 0.00000 0.05811 0.03108 0.03514 0.03378 0.03378 0.03378

65 Swellendam1 0.04459 0.03243 0.04324 0.00135 0.00270 0.00135 0.00135 0.00135

66 Tokai1 0.05811 0.00135 0.05946 0.03243 0.03649 0.03514 0.03514 0.03514

67 Uganda1 0.09459 0.08649 0.09595 0.08919 0.08784 0.08919 0.08919 0.08919

68 Villiersdorp1 0.04459 0.03243 0.04324 0.00135 0.00270 0.00135 0.00135 0.00135

69 Villiersdorp2 0.04459 0.03243 0.04324 0.00135 0.00270 0.00135 0.00135 0.00135

70 Villiersdorp3 0.04459 0.03243 0.04324 0.00135 0.00270 0.00135 0.00135 0.00135

71 Villiersdorp4 0.04459 0.03243 0.04324 0.00135 0.00270 0.00135 0.00135 0.00135

72 Villiersdorp5 0.04459 0.03243 0.04324 0.00135 0.00270 0.00135 0.00135 0.00135

73 KENYA 0.10270 0.10541 0.10405 0.10135 0.10000 0.10135 0.10135 0.10135

74 D.VARIEGATA 0.11317 0.09137 0.11770 0.09747 0.09900 0.10052 0.10052 0.10052

75 PortElizabeth 0.05359 0.03693 0.05353 0.01198 0.01347 0.01203 0.01203 0.01203

76 Wolkberg 0.06351 0.06622 0.06351 0.05811 0.06216 0.06081 0.06081 0.06081

77 Agulhas1 0.04730 0.03514 0.04595 0.00405 0.00000 0.00135 0.00135 0.00135

78 Agulhas2 0.05405 0.03919 0.05270 0.01081 0.00946 0.00811 0.00811 0.00811

79 Agulhas3 0.04730 0.03514 0.04595 0.00405 0.00000 0.00135 0.00135 0.00135

80 Bredasdorp1 0.05135 0.03649 0.05000 0.00811 0.00676 0.00541 0.00541 0.00541

81 Grahamstown1 0.05135 0.03514 0.05000 0.01081 0.01216 0.01081 0.01081 0.01081

82 Kleinmond1 0.04324 0.03108 0.04189 0.00000 0.00405 0.00270 0.00270 0.00270

83 Klipheuwel1 0.05811 0.00135 0.05946 0.03243 0.03649 0.03514 0.03514 0.03514

84 Klipheuwel2 0.05676 0.00000 0.05811 0.03108 0.03514 0.03378 0.03378 0.03378

85 SomersetWest8 0.05811 0.00135 0.05946 0.03243 0.03649 0.03514 0.03514 0.03514

86 Struisbaai1 0.05135 0.03649 0.05000 0.00811 0.00676 0.00541 0.00541 0.00541

87 Swellendam2 0.05405 0.03919 0.05270 0.01081 0.00946 0.00811 0.00811 0.00811

88 Swellendam3 0.05405 0.03919 0.05270 0.01081 0.00946 0.00811 0.00811 0.00811

89 Swellendam4 0.05135 0.03649 0.05000 0.00811 0.00676 0.00541 0.00541 0.00541

90 Swellendam5 0.05405 0.03919 0.05270 0.01081 0.00946 0.00811 0.00811 0.00811

91 Swellendam6 0.05405 0.03919 0.05270 0.01081 0.00946 0.00811 0.00811 0.00811

92 Swellendam7 0.05405 0.03919 0.05270 0.01081 0.00946 0.00811 0.00811 0.00811

Supplementary table 1 continues.

41 42 43 44 45 46 47 48

41 Napier5 -

42 Napier6 0.00135 -

43 NaturesValley1 0.00811 0.00676 -

44 Oudtshoorn1 0.00811 0.00676 0.01081 -

45 PortAlfred1 0.01081 0.00946 0.00811 0.01351 -

46 PortStJohns 0.04459 0.04324 0.04459 0.04865 0.05000 -

47 PringleBay1 0.00541 0.00405 0.00541 0.01081 0.01081 0.04189 -

48 PringleBay2 0.00405 0.00270 0.00405 0.00946 0.00946 0.04054 0.00135 -

49 PringleBay3 0.00135 0.00000 0.00676 0.00676 0.00946 0.04324 0.00405 0.00270

50 PringleBay4 0.00405 0.00270 0.00405 0.00946 0.00946 0.04054 0.00135 0.00000

51 Sabie1 0.03784 0.03649 0.03784 0.04324 0.04324 0.02568 0.03514 0.03378

52 Sabie2 0.03784 0.03649 0.03784 0.04324 0.04324 0.02568 0.03514 0.03378

53 Silvermine 0.00405 0.00270 0.00405 0.00946 0.00946 0.04054 0.00135 0.00000

54 SomersetWest1 0.03649 0.03514 0.03243 0.03919 0.03514 0.05811 0.03378 0.03243

55 SomersetWest2 0.03784 0.03649 0.03378 0.04054 0.03649 0.05946 0.03514 0.03378

56 SomersetWest3 0.03514 0.03378 0.03108 0.03784 0.03378 0.05676 0.03243 0.03108

57 SomersetWest4 0.03514 0.03378 0.03108 0.03784 0.03378 0.05676 0.03243 0.03108

58 SomersetWest5 0.03649 0.03514 0.03243 0.03919 0.03514 0.05811 0.03378 0.03243

59 SomersetWest6 0.03649 0.03514 0.03243 0.03919 0.03514 0.05811 0.03378 0.03243

60 SomersetWest7 0.03514 0.03378 0.03108 0.03784 0.03378 0.05676 0.03243 0.03108

61 Stellenbosch1 0.03514 0.03378 0.03108 0.03784 0.03378 0.05676 0.03243 0.03108

62 Stellenbosch2 0.03514 0.03378 0.03108 0.03784 0.03378 0.05676 0.03243 0.03108

63 Stellenbosch3 0.03514 0.03378 0.03108 0.03784 0.03378 0.05676 0.03243 0.03108

64 Stellenbosch4 0.03514 0.03378 0.03108 0.03784 0.03378 0.05676 0.03243 0.03108

65 Swellendam1 0.00270 0.00135 0.00541 0.00811 0.00811 0.04189 0.00270 0.00135

66 Tokai1 0.03649 0.03514 0.03243 0.03919 0.03514 0.05811 0.03378 0.03243

67 Uganda1 0.08784 0.08919 0.08919 0.08784 0.09054 0.09459 0.09054 0.08919

68 Villiersdorp1 0.00270 0.00135 0.00541 0.00811 0.00811 0.04189 0.00270 0.00135

69 Villiersdorp2 0.00270 0.00135 0.00541 0.00811 0.00811 0.04189 0.00270 0.00135

70 Villiersdorp3 0.00270 0.00135 0.00541 0.00811 0.00811 0.04189 0.00270 0.00135

71 Villiersdorp4 0.00270 0.00135 0.00541 0.00811 0.00811 0.04189 0.00270 0.00135

72 Villiersdorp5 0.00270 0.00135 0.00541 0.00811 0.00811 0.04189 0.00270 0.00135

73 KENYA 0.10000 0.10135 0.10405 0.10541 0.10541 0.10270 0.10270 0.10135

74 D.VARIEGATA 0.09905 0.10052 0.09757 0.10372 0.09763 0.11617 0.09900 0.09747

75 PortElizabeth 0.01352 0.01203 0.01060 0.01681 0.00149 0.05205 0.01347 0.01198

76 Wolkberg 0.05946 0.06081 0.06216 0.06757 0.06757 0.06216 0.05946 0.05811

77 Agulhas1 0.00270 0.00135 0.00811 0.00811 0.01081 0.04459 0.00541 0.00405

78 Agulhas2 0.00946 0.00811 0.00946 0.00676 0.01486 0.05135 0.01216 0.01081

79 Agulhas3 0.00270 0.00135 0.00811 0.00811 0.01081 0.04459 0.00541 0.00405

80 Bredasdorp1 0.00676 0.00541 0.00946 0.00405 0.01216 0.04865 0.00946 0.00811

81 Grahamstown1 0.01216 0.01081 0.00946 0.01486 0.00135 0.04865 0.01216 0.01081

82 Kleinmond1 0.00405 0.00270 0.00405 0.00946 0.00946 0.04054 0.00135 0.00000

83 Klipheuwel1 0.03649 0.03514 0.03243 0.03919 0.03514 0.05811 0.03378 0.03243

84 Klipheuwel2 0.03514 0.03378 0.03108 0.03784 0.03378 0.05676 0.03243 0.03108

85 SomersetWest8 0.03649 0.03514 0.03243 0.03919 0.03514 0.05811 0.03378 0.03243

86 Struisbaai1 0.00676 0.00541 0.00946 0.00405 0.01216 0.04865 0.00946 0.00811

87 Swellendam2 0.00946 0.00811 0.00946 0.00676 0.01486 0.05135 0.01216 0.01081

88 Swellendam3 0.00946 0.00811 0.00946 0.00676 0.01486 0.05135 0.01216 0.01081

89 Swellendam4 0.00676 0.00541 0.00946 0.00405 0.01216 0.04865 0.00946 0.00811

90 Swellendam5 0.00946 0.00811 0.00946 0.00676 0.01486 0.05135 0.01216 0.01081

91 Swellendam6 0.00946 0.00811 0.00946 0.00676 0.01486 0.05135 0.01216 0.01081

92 Swellendam7 0.00946 0.00811 0.00946 0.00676 0.01486 0.05135 0.01216 0.01081

Supplementary table 1 continues.

49 50 51 52 53 54 55 56

49 PringleBay3 -

50 PringleBay4 0.00270 -

51 Sabie1 0.03649 0.03378 -

52 Sabie2 0.03649 0.03378 0.00000 -

53 Silvermine 0.00270 0.00000 0.03378 0.03378 -

54 SomersetWest1 0.03514 0.03243 0.05405 0.05405 0.03243 -

55 SomersetWest2 0.03649 0.03378 0.05541 0.05541 0.03378 0.00135 -

56 SomersetWest3 0.03378 0.03108 0.05270 0.05270 0.03108 0.00135 0.00270 -

57 SomersetWest4 0.03378 0.03108 0.05270 0.05270 0.03108 0.00135 0.00270 0.00000

58 SomersetWest5 0.03514 0.03243 0.05405 0.05405 0.03243 0.00000 0.00135 0.00135

59 SomersetWest6 0.03514 0.03243 0.05405 0.05405 0.03243 0.00000 0.00135 0.00135

60 SomersetWest7 0.03378 0.03108 0.05270 0.05270 0.03108 0.00135 0.00270 0.00000

61 Stellenbosch1 0.03378 0.03108 0.05270 0.05270 0.03108 0.00135 0.00270 0.00000

62 Stellenbosch2 0.03378 0.03108 0.05270 0.05270 0.03108 0.00135 0.00270 0.00000

63 Stellenbosch3 0.03378 0.03108 0.05270 0.05270 0.03108 0.00135 0.00270 0.00000

64 Stellenbosch4 0.03378 0.03108 0.05270 0.05270 0.03108 0.00135 0.00270 0.00000

65 Swellendam1 0.00135 0.00135 0.03514 0.03514 0.00135 0.03378 0.03514 0.03243

66 Tokai1 0.03514 0.03243 0.05405 0.05405 0.03243 0.00000 0.00135 0.00135

67 Uganda1 0.08919 0.08919 0.09730 0.09730 0.08919 0.08649 0.08784 0.08649

68 Villiersdorp1 0.00135 0.00135 0.03514 0.03514 0.00135 0.03378 0.03514 0.03243

69 Villiersdorp2 0.00135 0.00135 0.03514 0.03514 0.00135 0.03378 0.03514 0.03243

70 Villiersdorp3 0.00135 0.00135 0.03514 0.03514 0.00135 0.03378 0.03514 0.03243

71 Villiersdorp4 0.00135 0.00135 0.03514 0.03514 0.00135 0.03378 0.03514 0.03243

72 Villiersdorp5 0.00135 0.00135 0.03514 0.03514 0.00135 0.03378 0.03514 0.03243

73 KENYA 0.10135 0.10135 0.10135 0.10135 0.10135 0.10676 0.10811 0.10541

74 D.VARIEGATA 0.10052 0.09747 0.11107 0.11107 0.09747 0.09131 0.09126 0.09137

75 PortElizabeth 0.01203 0.01198 0.04433 0.04433 0.01198 0.03842 0.03842 0.03693

76 Wolkberg 0.06081 0.05811 0.06486 0.06486 0.05811 0.06757 0.06757 0.06622

77 Agulhas1 0.00135 0.00405 0.03784 0.03784 0.00405 0.03649 0.03784 0.03514

78 Agulhas2 0.00811 0.01081 0.04459 0.04459 0.01081 0.04054 0.04189 0.03919

79 Agulhas3 0.00135 0.00405 0.03784 0.03784 0.00405 0.03649 0.03784 0.03514

80 Bredasdorp1 0.00541 0.00811 0.04189 0.04189 0.00811 0.03784 0.03919 0.03649

81 Grahamstown1 0.01081 0.01081 0.04189 0.04189 0.01081 0.03649 0.03784 0.03514

82 Kleinmond1 0.00270 0.00000 0.03378 0.03378 0.00000 0.03243 0.03378 0.03108

83 Klipheuwel1 0.03514 0.03243 0.05270 0.05270 0.03243 0.00270 0.00405 0.00135

84 Klipheuwel2 0.03378 0.03108 0.05270 0.05270 0.03108 0.00135 0.00270 0.00000

85 SomersetWest8 0.03514 0.03243 0.05270 0.05270 0.03243 0.00270 0.00405 0.00135

86 Struisbaai1 0.00541 0.00811 0.04189 0.04189 0.00811 0.03784 0.03919 0.03649

87 Swellendam2 0.00811 0.01081 0.04459 0.04459 0.01081 0.04054 0.04189 0.03919

88 Swellendam3 0.00811 0.01081 0.04459 0.04459 0.01081 0.04054 0.04189 0.03919

89 Swellendam4 0.00541 0.00811 0.04189 0.04189 0.00811 0.03784 0.03919 0.03649

90 Swellendam5 0.00811 0.01081 0.04459 0.04459 0.01081 0.04054 0.04189 0.03919

91 Swellendam6 0.00811 0.01081 0.04459 0.04459 0.01081 0.04054 0.04189 0.03919

92 Swellendam7 0.00811 0.01081 0.04459 0.04459 0.01081 0.04054 0.04189 0.03919

Supplementary table 1 continues.

57 58 59 60 61 62 63 64

57 SomersetWest4 -

58 SomersetWest5 0.00135 -

59 SomersetWest6 0.00135 0.00000 -

60 SomersetWest7 0.00000 0.00135 0.00135 -

61 Stellenbosch1 0.00000 0.00135 0.00135 0.00000 -

62 Stellenbosch2 0.00000 0.00135 0.00135 0.00000 0.00000 -

63 Stellenbosch3 0.00000 0.00135 0.00135 0.00000 0.00000 0.00000 -

64 Stellenbosch4 0.00000 0.00135 0.00135 0.00000 0.00000 0.00000 0.00000 -

65 Swellendam1 0.03243 0.03378 0.03378 0.03243 0.03243 0.03243 0.03243 0.03243

66 Tokai1 0.00135 0.00000 0.00000 0.00135 0.00135 0.00135 0.00135 0.00135

67 Uganda1 0.08649 0.08649 0.08649 0.08649 0.08649 0.08649 0.08649 0.08649

68 Villiersdorp1 0.03243 0.03378 0.03378 0.03243 0.03243 0.03243 0.03243 0.03243

69 Villiersdorp2 0.03243 0.03378 0.03378 0.03243 0.03243 0.03243 0.03243 0.03243

70 Villiersdorp3 0.03243 0.03378 0.03378 0.03243 0.03243 0.03243 0.03243 0.03243

71 Villiersdorp4 0.03243 0.03378 0.03378 0.03243 0.03243 0.03243 0.03243 0.03243

72 Villiersdorp5 0.03243 0.03378 0.03378 0.03243 0.03243 0.03243 0.03243 0.03243

73 KENYA 0.10541 0.10676 0.10676 0.10541 0.10541 0.10541 0.10541 0.10541

74 D.VARIEGATA 0.09137 0.09131 0.09131 0.09137 0.09137 0.09137 0.09137 0.09137

75 PortElizabeth 0.03693 0.03842 0.03842 0.03693 0.03693 0.03693 0.03693 0.03693

76 Wolkberg 0.06622 0.06757 0.06757 0.06622 0.06622 0.06622 0.06622 0.06622

77 Agulhas1 0.03514 0.03649 0.03649 0.03514 0.03514 0.03514 0.03514 0.03514

78 Agulhas2 0.03919 0.04054 0.04054 0.03919 0.03919 0.03919 0.03919 0.03919

79 Agulhas3 0.03514 0.03649 0.03649 0.03514 0.03514 0.03514 0.03514 0.03514

80 Bredasdorp1 0.03649 0.03784 0.03784 0.03649 0.03649 0.03649 0.03649 0.03649

81 Grahamstown1 0.03514 0.03649 0.03649 0.03514 0.03514 0.03514 0.03514 0.03514

82 Kleinmond1 0.03108 0.03243 0.03243 0.03108 0.03108 0.03108 0.03108 0.03108

83 Klipheuwel1 0.00135 0.00270 0.00270 0.00135 0.00135 0.00135 0.00135 0.00135

84 Klipheuwel2 0.00000 0.00135 0.00135 0.00000 0.00000 0.00000 0.00000 0.00000

85 SomersetWest8 0.00135 0.00270 0.00270 0.00135 0.00135 0.00135 0.00135 0.00135

86 Struisbaai1 0.03649 0.03784 0.03784 0.03649 0.03649 0.03649 0.03649 0.03649

87 Swellendam2 0.03919 0.04054 0.04054 0.03919 0.03919 0.03919 0.03919 0.03919

88 Swellendam3 0.03919 0.04054 0.04054 0.03919 0.03919 0.03919 0.03919 0.03919

89 Swellendam4 0.03649 0.03784 0.03784 0.03649 0.03649 0.03649 0.03649 0.03649

90 Swellendam5 0.03919 0.04054 0.04054 0.03919 0.03919 0.03919 0.03919 0.03919

91 Swellendam6 0.03919 0.04054 0.04054 0.03919 0.03919 0.03919 0.03919 0.03919

92 Swellendam7 0.03919 0.04054 0.04054 0.03919 0.03919 0.03919 0.03919 0.03919

Supplementary table 1 continues.

65 66 67 68 69 70 71 72

65 Swellendam1 -

66 Tokai1 0.03378 -

67 Uganda1 0.08784 0.08649 -

68 Villiersdorp1 0.00000 0.03378 0.08784 -

69 Villiersdorp2 0.00000 0.03378 0.08784 0.00000 -

70 Villiersdorp3 0.00000 0.03378 0.08784 0.00000 0.00000 -

71 Villiersdorp4 0.00000 0.03378 0.08784 0.00000 0.00000 0.00000 -

72 Villiersdorp5 0.00000 0.03378 0.08784 0.00000 0.00000 0.00000 0.00000 -

73 KENYA 0.10000 0.10676 0.06622 0.10000 0.10000 0.10000 0.10000 0.10000

74 D.VARIEGATA 0.09900 0.09131 0.12098 0.09900 0.09900 0.09900 0.09900 0.09900

75 PortElizabeth 0.01053 0.03842 0.10131 0.01053 0.01053 0.01053 0.01053 0.01053

76 Wolkberg 0.05946 0.06757 0.10270 0.05946 0.05946 0.05946 0.05946 0.05946

77 Agulhas1 0.00270 0.03649 0.08784 0.00270 0.00270 0.00270 0.00270 0.00270

78 Agulhas2 0.00946 0.04054 0.09189 0.00946 0.00946 0.00946 0.00946 0.00946

79 Agulhas3 0.00270 0.03649 0.08784 0.00270 0.00270 0.00270 0.00270 0.00270

80 Bredasdorp1 0.00676 0.03784 0.08919 0.00676 0.00676 0.00676 0.00676 0.00676

81 Grahamstown1 0.00946 0.03649 0.09189 0.00946 0.00946 0.00946 0.00946 0.00946

82 Kleinmond1 0.00135 0.03243 0.08919 0.00135 0.00135 0.00135 0.00135 0.00135

83 Klipheuwel1 0.03378 0.00270 0.08784 0.03378 0.03378 0.03378 0.03378 0.03378

84 Klipheuwel2 0.03243 0.00135 0.08649 0.03243 0.03243 0.03243 0.03243 0.03243

85 SomersetWest8 0.03378 0.00270 0.08784 0.03378 0.03378 0.03378 0.03378 0.03378

86 Struisbaai1 0.00676 0.03784 0.08919 0.00676 0.00676 0.00676 0.00676 0.00676

87 Swellendam2 0.00946 0.04054 0.09189 0.00946 0.00946 0.00946 0.00946 0.00946

88 Swellendam3 0.00946 0.04054 0.09189 0.00946 0.00946 0.00946 0.00946 0.00946

89 Swellendam4 0.00676 0.03784 0.08919 0.00676 0.00676 0.00676 0.00676 0.00676

90 Swellendam5 0.00946 0.04054 0.09189 0.00946 0.00946 0.00946 0.00946 0.00946

91 Swellendam6 0.00946 0.04054 0.09189 0.00946 0.00946 0.00946 0.00946 0.00946

92 Swellendam7 0.00946 0.04054 0.09189 0.00946 0.00946 0.00946 0.00946 0.00946

Supplementary table 1 continues.

73 74 75 76 77 78 79 80

73 KENYA -

74 D.VARIEGATA 0.11887 -

75 PortElizabeth 0.09784 0.09955 -

76 Wolkberg 0.10135 0.10551 0.06959 -

77 Agulhas1 0.10000 0.09900 0.01347 0.06216 -

78 Agulhas2 0.10676 0.10403 0.01835 0.06892 0.00946 -

79 Agulhas3 0.10000 0.09900 0.01347 0.06216 0.00000 0.00946 -

80 Bredasdorp1 0.10405 0.10068 0.01513 0.06622 0.00676 0.00270 0.00676 -

81 Grahamstown1 0.10405 0.09910 0.00000 0.06622 0.01216 0.01622 0.01216 0.01351

82 Kleinmond1 0.10135 0.09747 0.01198 0.05811 0.00405 0.01081 0.00405 0.00811

83 Klipheuwel1 0.10676 0.09289 0.03842 0.06757 0.03649 0.04054 0.03649 0.03784

84 Klipheuwel2 0.10541 0.09137 0.03693 0.06622 0.03514 0.03919 0.03514 0.03649

85 SomersetWest8 0.10676 0.09289 0.03842 0.06757 0.03649 0.04054 0.03649 0.03784

86 Struisbaai1 0.10405 0.10068 0.01513 0.06622 0.00676 0.00270 0.00676 0.00000

87 Swellendam2 0.10676 0.10403 0.01835 0.06892 0.00946 0.00000 0.00946 0.00270

88 Swellendam3 0.10676 0.10403 0.01835 0.06892 0.00946 0.00000 0.00946 0.00270

89 Swellendam4 0.10405 0.10068 0.01513 0.06622 0.00676 0.00270 0.00676 0.00000

90 Swellendam5 0.10676 0.10403 0.01835 0.06892 0.00946 0.00000 0.00946 0.00270

91 Swellendam6 0.10676 0.10403 0.01835 0.06892 0.00946 0.00000 0.00946 0.00270

92 Swellendam7 0.10676 0.10403 0.01835 0.06892 0.00946 0.00000 0.00946 0.00270

Supplementary table 1 continues.

81 82 83 84 85 86 87 88

81 Grahamstown1 -

82 Kleinmond1 0.01081 -

83 Klipheuwel1 0.03649 0.03243 -

84 Klipheuwel2 0.03514 0.03108 0.00135 -

85 SomersetWest8 0.03649 0.03243 0.00000 0.00135 -

86 Struisbaai1 0.01351 0.00811 0.03784 0.03649 0.03784 -

87 Swellendam2 0.01622 0.01081 0.04054 0.03919 0.04054 0.00270 -

88 Swellendam3 0.01622 0.01081 0.04054 0.03919 0.04054 0.00270 0.00000 -

89 Swellendam4 0.01351 0.00811 0.03784 0.03649 0.03784 0.00000 0.00270 0.00270

90 Swellendam5 0.01622 0.01081 0.04054 0.03919 0.04054 0.00270 0.00000 0.00000

91 Swellendam6 0.01622 0.01081 0.04054 0.03919 0.04054 0.00270 0.00000 0.00000

92 Swellendam7 0.01622 0.01081 0.04054 0.03919 0.04054 0.00270 0.00000 0.00000

Supplementary table 1 continues.

89 90 91 92

89 Swellendam4 -

90 Swellendam5 0.00270 -

91 Swellendam6 0.00270 0.00000 -

92 Swellendam7 0.00270 0.00000 0.00000 -
