# Supplementary material for: Molecular evidence for cryptic species in the common slug eating snake Duberrialutrixlutrix (Squamata, Lamprophiidae) from South Africa
Source: Zookeys. 2019 Apr 15;838:133–54. doi: 10.3897/zookeys.838.32022 (PMC6477839; doi:10.3897/zookeys.838.32022)
Supplement: Supplementary material 2 [file zookeys-838-133-s002.docx]

Supplementary table 2 continues. Uncorrected “p” distances for the cyt *b* for the *Duberria lutrix lutrix* sampled during the present study.

1 2 3 4 5 6 7 8

1 A.mltmclts -

2 D.vivax 0.17869 -

3 D.variegata 0.18377 0.20447 -

4 Agulhas1 0.18689 0.20000 0.09774 -

5 Agulhas2 0.18197 0.19836 0.09262 0.00492 -

6 Agulhas3 0.18689 0.20000 0.09774 0.00000 0.00492 -

7 Ashton1 0.18361 0.19836 0.09262 0.00492 0.00328 0.00492 -

8 Ashton2 0.18361 0.19836 0.09262 0.00492 0.00328 0.00492 0.00000 -

9 Ashton3 0.18395 0.19875 0.09101 0.00492 0.00328 0.00492 0.00000 0.00000

10 Ashton4 0.18361 0.19836 0.09262 0.00492 0.00328 0.00492 0.00000 0.00000

11 Ashton5 0.18361 0.19836 0.09262 0.00492 0.00328 0.00492 0.00000 0.00000

12 Ashton6 0.18361 0.19836 0.09262 0.00492 0.00328 0.00492 0.00000 0.00000

13 Ashton7 0.18361 0.19836 0.09262 0.00492 0.00328 0.00492 0.00000 0.00000

14 Ashton8 0.18361 0.19836 0.09262 0.00492 0.00328 0.00492 0.00000 0.00000

15 Bergvliet1 0.18525 0.20000 0.10638 0.02131 0.02295 0.02131 0.02295 0.02295

16 Bredasdorp1 0.18361 0.20000 0.09427 0.00328 0.00164 0.00328 0.00164 0.00164

17 Caledon1 0.18705 0.20021 0.09786 0.00328 0.00492 0.00328 0.00492 0.00492

18 Entabeni1 0.18525 0.20164 0.10652 0.06066 0.06066 0.06066 0.06066 0.06066

19 Flakkenberg1 0.18525 0.20000 0.10638 0.02131 0.02295 0.02131 0.02295 0.02295

20 Genadendal1 0.18689 0.19672 0.09767 0.00328 0.00492 0.00328 0.00492 0.00492

21 Grahamnstown1 0.19016 0.20164 0.10290 0.01148 0.01311 0.01148 0.01311 0.01311

22 Greyton1 0.18689 0.19672 0.09767 0.00328 0.00492 0.00328 0.00492 0.00492

23 Greyton2 0.18689 0.19672 0.09767 0.00328 0.00492 0.00328 0.00492 0.00492

24 Greyton3 0.18689 0.19672 0.09767 0.00328 0.00492 0.00328 0.00492 0.00492

25 Herbertsdale1 0.18361 0.20000 0.09427 0.00328 0.00164 0.00328 0.00164 0.00164

26 HighWaters1 0.17869 0.19672 0.09625 0.03443 0.03115 0.03443 0.03443 0.03443

27 HopeFountain1 0.19016 0.20164 0.10290 0.01148 0.01311 0.01148 0.01311 0.01311

28 Humansdorp1 0.18689 0.19672 0.09949 0.00656 0.00820 0.00656 0.00820 0.00820

29 Jacobsbaai1 0.18689 0.19672 0.09767 0.00328 0.00492 0.00328 0.00492 0.00492

30 Kirstenbosch1 0.18525 0.20000 0.10638 0.02131 0.02295 0.02131 0.02295 0.02295

31 Kirstenbosch2 0.18525 0.20000 0.10638 0.02131 0.02295 0.02131 0.02295 0.02295

32 Kirstenbosch3 0.18525 0.20000 0.10638 0.02131 0.02295 0.02131 0.02295 0.02295

33 Kleinmond1 0.18525 0.19836 0.09938 0.00492 0.00656 0.00492 0.00656 0.00656

34 Klipheuwel1 0.18525 0.20000 0.10638 0.02131 0.02295 0.02131 0.02295 0.02295

35 Klipheuwel2 0.18525 0.20000 0.10638 0.02131 0.02295 0.02131 0.02295 0.02295

36 Kokstad1 0.17869 0.20000 0.09624 0.03770 0.03443 0.03770 0.03770 0.03770

37 Kokstad2 0.17869 0.20000 0.09624 0.03770 0.03443 0.03770 0.03770 0.03770

38 Kokstad3 0.18033 0.19836 0.09453 0.03607 0.03279 0.03607 0.03607 0.03607

39 Kokstad4 0.17869 0.20000 0.09624 0.03770 0.03443 0.03770 0.03770 0.03770

40 Kokstad5 0.17869 0.20000 0.09624 0.03770 0.03443 0.03770 0.03770 0.03770

41 Kokstad6 0.17869 0.19836 0.09454 0.03443 0.03115 0.03443 0.03443 0.03443

42 Kokstad7 0.17869 0.20000 0.09624 0.03770 0.03443 0.03770 0.03770 0.03770

43 Kraaifontein1 0.18361 0.20164 0.10473 0.02295 0.02459 0.02295 0.02459 0.02459

44 Kwancele1 0.17869 0.19836 0.09454 0.03443 0.03115 0.03443 0.03443 0.03443

45 LakesideCT1 0.18852 0.19836 0.09764 0.00492 0.00656 0.00492 0.00656 0.00656

46 Napier1 0.18525 0.20000 0.09598 0.00328 0.00328 0.00328 0.00328 0.00328

47 Napier2 0.18525 0.19836 0.09948 0.00492 0.00656 0.00492 0.00656 0.00656

48 Napier3 0.18689 0.20000 0.09773 0.00328 0.00492 0.00328 0.00492 0.00492

49 Napier4 0.18852 0.20000 0.09780 0.00656 0.00820 0.00656 0.00492 0.00492

50 Napier5 0.18689 0.20000 0.09773 0.00328 0.00492 0.00328 0.00492 0.00492

51 Napier6 0.18689 0.20000 0.09773 0.00328 0.00492 0.00328 0.00492 0.00492

52 NaturesValley1 0.19180 0.20328 0.10291 0.00984 0.01148 0.00984 0.01148 0.01148

53 Oudtdhoorn1 0.18525 0.20164 0.09600 0.00492 0.00328 0.00492 0.00328 0.00328

54 PortAlfred1 0.18361 0.19508 0.09784 0.00820 0.00984 0.00820 0.00984 0.00984

55 PortStJohns1 0.17869 0.19836 0.09454 0.03443 0.03115 0.03443 0.03443 0.03443

56 PringleBay1 0.18689 0.19672 0.09767 0.00328 0.00492 0.00328 0.00492 0.00492

57 PringleBay2 0.18525 0.19836 0.09938 0.00492 0.00656 0.00492 0.00656 0.00656

58 PringleBay3 0.18689 0.20000 0.09773 0.00328 0.00492 0.00328 0.00492 0.00492

59 PringleBay4 0.18525 0.19836 0.09938 0.00492 0.00656 0.00492 0.00656 0.00656

60 Sabie1 0.18689 0.19180 0.09628 0.03934 0.03607 0.03934 0.03934 0.03934

61 Sabie2 0.18525 0.19344 0.09793 0.04098 0.03770 0.04098 0.04098 0.04098

62 Silvermine1 0.18852 0.19672 0.09594 0.00492 0.00656 0.00492 0.00656 0.00656

63 SomersetWest1 0.18525 0.20000 0.10638 0.02131 0.02295 0.02131 0.02295 0.02295

64 SomersetWest2 0.18525 0.20000 0.10638 0.02131 0.02295 0.02131 0.02295 0.02295

65 SomersetWest3 0.18525 0.20000 0.10638 0.02131 0.02295 0.02131 0.02295 0.02295

66 SomersetWest4 0.18525 0.20000 0.10638 0.02131 0.02295 0.02131 0.02295 0.02295

67 SomersetWest5 0.18525 0.20000 0.10638 0.02131 0.02295 0.02131 0.02295 0.02295

68 SomersetWest6 0.18525 0.20000 0.10638 0.02131 0.02295 0.02131 0.02295 0.02295

69 SomersetWest7 0.18689 0.20164 0.10803 0.02295 0.02459 0.02295 0.02459 0.02459

70 SomersetWest8 0.18525 0.20000 0.10638 0.02131 0.02295 0.02131 0.02295 0.02295

71 Stellenbosch1 0.18525 0.20000 0.10638 0.02131 0.02295 0.02131 0.02295 0.02295

72 Stellenbosch2 0.18525 0.20000 0.10638 0.02131 0.02295 0.02131 0.02295 0.02295

73 Stellenbosch3 0.18525 0.20000 0.10638 0.02131 0.02295 0.02131 0.02295 0.02295

74 Stellenbosch4 0.18525 0.20000 0.10638 0.02131 0.02295 0.02131 0.02295 0.02295

75 Struisbaai1 0.18361 0.20000 0.09427 0.00328 0.00164 0.00328 0.00164 0.00164

76 Swellendam1 0.18689 0.19672 0.09767 0.00328 0.00492 0.00328 0.00492 0.00492

77 Swellendam2 0.18197 0.19836 0.09262 0.00492 0.00000 0.00492 0.00328 0.00328

78 Swellendam3 0.18197 0.19836 0.09262 0.00492 0.00000 0.00492 0.00328 0.00328

79 Swellendam4 0.18361 0.20000 0.09427 0.00328 0.00164 0.00328 0.00164 0.00164

80 Swellendam5 0.18197 0.19836 0.09262 0.00492 0.00000 0.00492 0.00328 0.00328

81 Swellendam6 0.18197 0.19836 0.09262 0.00492 0.00000 0.00492 0.00328 0.00328

82 Swellendam7 0.18197 0.19836 0.09262 0.00492 0.00000 0.00492 0.00328 0.00328

83 Tokai1 0.18525 0.20000 0.10638 0.02131 0.02295 0.02131 0.02295 0.02295

84 Uganda1 0.19180 0.20492 0.12705 0.10000 0.09508 0.10000 0.09836 0.09836

85 Villiersdorp1 0.18689 0.19672 0.09767 0.00328 0.00492 0.00328 0.00492 0.00492

86 Villiersdorp2 0.18689 0.19672 0.09767 0.00328 0.00492 0.00328 0.00492 0.00492

87 Villiersdorp3 0.18689 0.19672 0.09767 0.00328 0.00492 0.00328 0.00492 0.00492

88 Villiersdorp4 0.18689 0.19672 0.09767 0.00328 0.00492 0.00328 0.00492 0.00492

89 Villiersdorp5 0.18689 0.19672 0.09767 0.00328 0.00492 0.00328 0.00492 0.00492

90 Wolkberg1 0.18689 0.20164 0.10468 0.06393 0.06393 0.06393 0.06393 0.06393

91 Kenya1 0.19344 0.19672 0.12373 0.09016 0.08689 0.09016 0.08689 0.08689

92 PortElizabeth 0.18052 0.20273 0.09182 0.00894 0.01123 0.00894 0.00896 0.00896

Supplementary table 2 continues.

9 10 11 12 13 14 15 16

9 Ashton3 -

10 Ashton4 0.00000 -

11 Ashton5 0.00000 0.00000 -

12 Ashton6 0.00000 0.00000 0.00000 -

13 Ashton7 0.00000 0.00000 0.00000 0.00000 -

14 Ashton8 0.00000 0.00000 0.00000 0.00000 0.00000 -

15 Bergvliet1 0.02298 0.02295 0.02295 0.02295 0.02295 0.02295 -

16 Bredasdorp1 0.00164 0.00164 0.00164 0.00164 0.00164 0.00164 0.02131 -

17 Caledon1 0.00492 0.00492 0.00492 0.00492 0.00492 0.00492 0.02133 0.00328

18 Entabeni1 0.05908 0.06066 0.06066 0.06066 0.06066 0.06066 0.06230 0.05902

19 Flakkenberg1 0.02298 0.02295 0.02295 0.02295 0.02295 0.02295 0.00000 0.02131

20 Genadendal1 0.00492 0.00492 0.00492 0.00492 0.00492 0.00492 0.02131 0.00328

21 Grahamnstown1 0.01311 0.01311 0.01311 0.01311 0.01311 0.01311 0.02623 0.01148

22 Greyton1 0.00492 0.00492 0.00492 0.00492 0.00492 0.00492 0.02131 0.00328

23 Greyton2 0.00492 0.00492 0.00492 0.00492 0.00492 0.00492 0.02131 0.00328

24 Greyton3 0.00492 0.00492 0.00492 0.00492 0.00492 0.00492 0.02131 0.00328

25 Herbertsdale1 0.00164 0.00164 0.00164 0.00164 0.00164 0.00164 0.02131 0.00000

26 HighWaters1 0.03281 0.03443 0.03443 0.03443 0.03443 0.03443 0.03607 0.03279

27 HopeFountain1 0.01311 0.01311 0.01311 0.01311 0.01311 0.01311 0.02623 0.01148

28 Humansdorp1 0.00820 0.00820 0.00820 0.00820 0.00820 0.00820 0.02131 0.00656

29 Jacobsbaai1 0.00492 0.00492 0.00492 0.00492 0.00492 0.00492 0.02131 0.00328

30 Kirstenbosch1 0.02298 0.02295 0.02295 0.02295 0.02295 0.02295 0.00000 0.02131

31 Kirstenbosch2 0.02298 0.02295 0.02295 0.02295 0.02295 0.02295 0.00000 0.02131

32 Kirstenbosch3 0.02298 0.02295 0.02295 0.02295 0.02295 0.02295 0.00000 0.02131

33 Kleinmond1 0.00656 0.00656 0.00656 0.00656 0.00656 0.00656 0.01967 0.00492

34 Klipheuwel1 0.02298 0.02295 0.02295 0.02295 0.02295 0.02295 0.00000 0.02131

35 Klipheuwel2 0.02298 0.02295 0.02295 0.02295 0.02295 0.02295 0.00000 0.02131

36 Kokstad1 0.03610 0.03770 0.03770 0.03770 0.03770 0.03770 0.03934 0.03607

37 Kokstad2 0.03610 0.03770 0.03770 0.03770 0.03770 0.03770 0.03934 0.03607

38 Kokstad3 0.03445 0.03607 0.03607 0.03607 0.03607 0.03607 0.03770 0.03443

39 Kokstad4 0.03610 0.03770 0.03770 0.03770 0.03770 0.03770 0.03934 0.03607

40 Kokstad5 0.03610 0.03770 0.03770 0.03770 0.03770 0.03770 0.03934 0.03607

41 Kokstad6 0.03282 0.03443 0.03443 0.03443 0.03443 0.03443 0.03934 0.03279

42 Kokstad7 0.03610 0.03770 0.03770 0.03770 0.03770 0.03770 0.03934 0.03607

43 Kraaifontein1 0.02462 0.02459 0.02459 0.02459 0.02459 0.02459 0.00164 0.02295

44 Kwancele1 0.03282 0.03443 0.03443 0.03443 0.03443 0.03443 0.03934 0.03279

45 LakesideCT1 0.00656 0.00656 0.00656 0.00656 0.00656 0.00656 0.02295 0.00492

46 Napier1 0.00328 0.00328 0.00328 0.00328 0.00328 0.00328 0.02131 0.00164

47 Napier2 0.00657 0.00656 0.00656 0.00656 0.00656 0.00656 0.02295 0.00492

48 Napier3 0.00492 0.00492 0.00492 0.00492 0.00492 0.00492 0.02131 0.00328

49 Napier4 0.00492 0.00492 0.00492 0.00492 0.00492 0.00492 0.02131 0.00656

50 Napier5 0.00492 0.00492 0.00492 0.00492 0.00492 0.00492 0.02131 0.00328

51 Napier6 0.00492 0.00492 0.00492 0.00492 0.00492 0.00492 0.02131 0.00328

52 NaturesValley1 0.01148 0.01148 0.01148 0.01148 0.01148 0.01148 0.02131 0.00984

53 Oudtdhoorn1 0.00328 0.00328 0.00328 0.00328 0.00328 0.00328 0.02295 0.00164

54 PortAlfred1 0.00984 0.00984 0.00984 0.00984 0.00984 0.00984 0.02295 0.00820

55 PortStJohns1 0.03282 0.03443 0.03443 0.03443 0.03443 0.03443 0.03934 0.03279

56 PringleBay1 0.00492 0.00492 0.00492 0.00492 0.00492 0.00492 0.02131 0.00328

57 PringleBay2 0.00656 0.00656 0.00656 0.00656 0.00656 0.00656 0.01967 0.00492

58 PringleBay3 0.00492 0.00492 0.00492 0.00492 0.00492 0.00492 0.02131 0.00328

59 PringleBay4 0.00656 0.00656 0.00656 0.00656 0.00656 0.00656 0.01967 0.00492

60 Sabie1 0.03775 0.03934 0.03934 0.03934 0.03934 0.03934 0.03770 0.03770

61 Sabie2 0.03939 0.04098 0.04098 0.04098 0.04098 0.04098 0.03934 0.03934

62 Silvermine1 0.00656 0.00656 0.00656 0.00656 0.00656 0.00656 0.02295 0.00492

63 SomersetWest1 0.02298 0.02295 0.02295 0.02295 0.02295 0.02295 0.00000 0.02131

64 SomersetWest2 0.02298 0.02295 0.02295 0.02295 0.02295 0.02295 0.00000 0.02131

65 SomersetWest3 0.02298 0.02295 0.02295 0.02295 0.02295 0.02295 0.00000 0.02131

66 SomersetWest4 0.02298 0.02295 0.02295 0.02295 0.02295 0.02295 0.00000 0.02131

67 SomersetWest5 0.02298 0.02295 0.02295 0.02295 0.02295 0.02295 0.00000 0.02131

68 SomersetWest6 0.02298 0.02295 0.02295 0.02295 0.02295 0.02295 0.00000 0.02131

69 SomersetWest7 0.02462 0.02459 0.02459 0.02459 0.02459 0.02459 0.00164 0.02295

70 SomersetWest8 0.02298 0.02295 0.02295 0.02295 0.02295 0.02295 0.00000 0.02131

71 Stellenbosch1 0.02298 0.02295 0.02295 0.02295 0.02295 0.02295 0.00000 0.02131

72 Stellenbosch2 0.02298 0.02295 0.02295 0.02295 0.02295 0.02295 0.00000 0.02131

73 Stellenbosch3 0.02298 0.02295 0.02295 0.02295 0.02295 0.02295 0.00000 0.02131

74 Stellenbosch4 0.02298 0.02295 0.02295 0.02295 0.02295 0.02295 0.00000 0.02131

75 Struisbaai1 0.00164 0.00164 0.00164 0.00164 0.00164 0.00164 0.02131 0.00000

76 Swellendam1 0.00492 0.00492 0.00492 0.00492 0.00492 0.00492 0.02131 0.00328

77 Swellendam2 0.00328 0.00328 0.00328 0.00328 0.00328 0.00328 0.02295 0.00164

78 Swellendam3 0.00328 0.00328 0.00328 0.00328 0.00328 0.00328 0.02295 0.00164

79 Swellendam4 0.00164 0.00164 0.00164 0.00164 0.00164 0.00164 0.02131 0.00000

80 Swellendam5 0.00328 0.00328 0.00328 0.00328 0.00328 0.00328 0.02295 0.00164

81 Swellendam6 0.00328 0.00328 0.00328 0.00328 0.00328 0.00328 0.02295 0.00164

82 Swellendam7 0.00328 0.00328 0.00328 0.00328 0.00328 0.00328 0.02295 0.00164

83 Tokai1 0.02298 0.02295 0.02295 0.02295 0.02295 0.02295 0.00000 0.02131

84 Uganda1 0.09681 0.09836 0.09836 0.09836 0.09836 0.09836 0.10820 0.09672

85 Villiersdorp1 0.00492 0.00492 0.00492 0.00492 0.00492 0.00492 0.02131 0.00328

86 Villiersdorp2 0.00492 0.00492 0.00492 0.00492 0.00492 0.00492 0.02131 0.00328

87 Villiersdorp3 0.00492 0.00492 0.00492 0.00492 0.00492 0.00492 0.02131 0.00328

88 Villiersdorp4 0.00492 0.00492 0.00492 0.00492 0.00492 0.00492 0.02131 0.00328

89 Villiersdorp5 0.00492 0.00492 0.00492 0.00492 0.00492 0.00492 0.02131 0.00328

90 Wolkberg1 0.06239 0.06393 0.06393 0.06393 0.06393 0.06393 0.05902 0.06230

91 Kenya1 0.08530 0.08689 0.08689 0.08689 0.08689 0.08689 0.10164 0.08852

92 PortElizabeth 0.00896 0.00896 0.00896 0.00896 0.00896 0.00896 0.02654 0.00894

Supplementary table 2 continues.

17 18 19 20 21 22 23 24

17 Caledon1 -

18 Entabeni1 0.06077 -

19 Flakkenberg1 0.02133 0.06230 -

20 Genadendal1 0.00330 0.06066 0.02131 -

21 Grahamnstown1 0.01151 0.05902 0.02623 0.01148 -

22 Greyton1 0.00330 0.06066 0.02131 0.00000 0.01148 -

23 Greyton2 0.00330 0.06066 0.02131 0.00000 0.01148 0.00000 -

24 Greyton3 0.00330 0.06066 0.02131 0.00000 0.01148 0.00000 0.00000 -

25 Herbertsdale1 0.00328 0.05902 0.02131 0.00328 0.01148 0.00328 0.00328 0.00328

26 HighWaters1 0.03448 0.06066 0.03607 0.03443 0.03607 0.03443 0.03443 0.03443

27 HopeFountain1 0.01151 0.05902 0.02623 0.01148 0.00000 0.01148 0.01148 0.01148

28 Humansdorp1 0.00656 0.05738 0.02131 0.00656 0.00492 0.00656 0.00656 0.00656

29 Jacobsbaai1 0.00330 0.06066 0.02131 0.00000 0.01148 0.00000 0.00000 0.00000

30 Kirstenbosch1 0.02133 0.06230 0.00000 0.02131 0.02623 0.02131 0.02131 0.02131

31 Kirstenbosch2 0.02133 0.06230 0.00000 0.02131 0.02623 0.02131 0.02131 0.02131

32 Kirstenbosch3 0.02133 0.06230 0.00000 0.02131 0.02623 0.02131 0.02131 0.02131

33 Kleinmond1 0.00494 0.05902 0.01967 0.00164 0.01311 0.00164 0.00164 0.00164

34 Klipheuwel1 0.02133 0.06230 0.00000 0.02131 0.02623 0.02131 0.02131 0.02131

35 Klipheuwel2 0.02133 0.06230 0.00000 0.02131 0.02623 0.02131 0.02131 0.02131

36 Kokstad1 0.03778 0.06393 0.03934 0.03770 0.03934 0.03770 0.03770 0.03770

37 Kokstad2 0.03778 0.06393 0.03934 0.03770 0.03934 0.03770 0.03770 0.03770

38 Kokstad3 0.03614 0.06230 0.03770 0.03607 0.03770 0.03607 0.03607 0.03607

39 Kokstad4 0.03778 0.06393 0.03934 0.03770 0.03934 0.03770 0.03770 0.03770

40 Kokstad5 0.03778 0.06393 0.03934 0.03770 0.03934 0.03770 0.03770 0.03770

41 Kokstad6 0.03448 0.06230 0.03934 0.03443 0.03607 0.03443 0.03443 0.03443

42 Kokstad7 0.03778 0.06393 0.03934 0.03770 0.03934 0.03770 0.03770 0.03770

43 Kraaifontein1 0.02297 0.06393 0.00164 0.02295 0.02787 0.02295 0.02295 0.02295

44 Kwancele1 0.03448 0.06230 0.03934 0.03443 0.03607 0.03443 0.03443 0.03443

45 LakesideCT1 0.00494 0.06230 0.02295 0.00164 0.01311 0.00164 0.00164 0.00164

46 Napier1 0.00328 0.05738 0.02131 0.00328 0.01148 0.00328 0.00328 0.00328

47 Napier2 0.00164 0.06230 0.02295 0.00492 0.01311 0.00492 0.00492 0.00492

48 Napier3 0.00000 0.06066 0.02131 0.00328 0.01148 0.00328 0.00328 0.00328

49 Napier4 0.00328 0.06393 0.02131 0.00656 0.01475 0.00656 0.00656 0.00656

50 Napier5 0.00000 0.06066 0.02131 0.00328 0.01148 0.00328 0.00328 0.00328

51 Napier6 0.00000 0.06066 0.02131 0.00328 0.01148 0.00328 0.00328 0.00328

52 NaturesValley1 0.00984 0.06066 0.02131 0.00984 0.00820 0.00984 0.00984 0.00984

53 Oudtdhoorn1 0.00492 0.06066 0.02295 0.00492 0.01311 0.00492 0.00492 0.00492

54 PortAlfred1 0.00820 0.05246 0.02295 0.00820 0.00656 0.00820 0.00820 0.00820

55 PortStJohns1 0.03448 0.06230 0.03934 0.03443 0.03607 0.03443 0.03443 0.03443

56 PringleBay1 0.00330 0.06066 0.02131 0.00000 0.01148 0.00000 0.00000 0.00000

57 PringleBay2 0.00494 0.05902 0.01967 0.00164 0.01311 0.00164 0.00164 0.00164

58 PringleBay3 0.00000 0.06066 0.02131 0.00328 0.01148 0.00328 0.00328 0.00328

59 PringleBay4 0.00494 0.05902 0.01967 0.00164 0.01311 0.00164 0.00164 0.00164

60 Sabie1 0.03940 0.06721 0.03770 0.03934 0.04098 0.03934 0.03934 0.03934

61 Sabie2 0.04106 0.06885 0.03934 0.04098 0.04262 0.04098 0.04098 0.04098

62 Silvermine1 0.00495 0.06230 0.02295 0.00164 0.01311 0.00164 0.00164 0.00164

63 SomersetWest1 0.02133 0.06230 0.00000 0.02131 0.02623 0.02131 0.02131 0.02131

64 SomersetWest2 0.02133 0.06230 0.00000 0.02131 0.02623 0.02131 0.02131 0.02131

65 SomersetWest3 0.02133 0.06230 0.00000 0.02131 0.02623 0.02131 0.02131 0.02131

66 SomersetWest4 0.02133 0.06230 0.00000 0.02131 0.02623 0.02131 0.02131 0.02131

67 SomersetWest5 0.02133 0.06230 0.00000 0.02131 0.02623 0.02131 0.02131 0.02131

68 SomersetWest6 0.02133 0.06230 0.00000 0.02131 0.02623 0.02131 0.02131 0.02131

69 SomersetWest7 0.02299 0.06393 0.00164 0.02295 0.02787 0.02295 0.02295 0.02295

70 SomersetWest8 0.02133 0.06230 0.00000 0.02131 0.02623 0.02131 0.02131 0.02131

71 Stellenbosch1 0.02133 0.06230 0.00000 0.02131 0.02623 0.02131 0.02131 0.02131

72 Stellenbosch2 0.02133 0.06230 0.00000 0.02131 0.02623 0.02131 0.02131 0.02131

73 Stellenbosch3 0.02133 0.06230 0.00000 0.02131 0.02623 0.02131 0.02131 0.02131

74 Stellenbosch4 0.02133 0.06230 0.00000 0.02131 0.02623 0.02131 0.02131 0.02131

75 Struisbaai1 0.00328 0.05902 0.02131 0.00328 0.01148 0.00328 0.00328 0.00328

76 Swellendam1 0.00330 0.06066 0.02131 0.00000 0.01148 0.00000 0.00000 0.00000

77 Swellendam2 0.00492 0.06066 0.02295 0.00492 0.01311 0.00492 0.00492 0.00492

78 Swellendam3 0.00492 0.06066 0.02295 0.00492 0.01311 0.00492 0.00492 0.00492

79 Swellendam4 0.00328 0.05902 0.02131 0.00328 0.01148 0.00328 0.00328 0.00328

80 Swellendam5 0.00492 0.06066 0.02295 0.00492 0.01311 0.00492 0.00492 0.00492

81 Swellendam6 0.00492 0.06066 0.02295 0.00492 0.01311 0.00492 0.00492 0.00492

82 Swellendam7 0.00492 0.06066 0.02295 0.00492 0.01311 0.00492 0.00492 0.00492

83 Tokai1 0.02133 0.06230 0.00000 0.02131 0.02623 0.02131 0.02131 0.02131

84 Uganda1 0.10022 0.10984 0.10820 0.10000 0.09836 0.10000 0.10000 0.10000

85 Villiersdorp1 0.00330 0.06066 0.02131 0.00000 0.01148 0.00000 0.00000 0.00000

86 Villiersdorp2 0.00330 0.06066 0.02131 0.00000 0.01148 0.00000 0.00000 0.00000

87 Villiersdorp3 0.00330 0.06066 0.02131 0.00000 0.01148 0.00000 0.00000 0.00000

88 Villiersdorp4 0.00330 0.06066 0.02131 0.00000 0.01148 0.00000 0.00000 0.00000

89 Villiersdorp5 0.00330 0.06066 0.02131 0.00000 0.01148 0.00000 0.00000 0.00000

90 Wolkberg1 0.06404 0.01311 0.05902 0.06393 0.06557 0.06393 0.06393 0.06393

91 Kenya1 0.09037 0.09836 0.10164 0.08689 0.09016 0.08689 0.08689 0.08689

92 PortElizabeth 0.00892 0.04915 0.02654 0.00663 0.00000 0.00663 0.00663 0.00663

Supplementary table 2 continues.

25 26 27 28 29 30 31 32

25 Herbertsdale1 -

26 HighWaters1 0.03279 -

27 HopeFountain1 0.01148 0.03607 -

28 Humansdorp1 0.00656 0.03115 0.00492 -

29 Jacobsbaai1 0.00328 0.03443 0.01148 0.00656 -

30 Kirstenbosch1 0.02131 0.03607 0.02623 0.02131 0.02131 -

31 Kirstenbosch2 0.02131 0.03607 0.02623 0.02131 0.02131 0.00000 -

32 Kirstenbosch3 0.02131 0.03607 0.02623 0.02131 0.02131 0.00000 0.00000 -

33 Kleinmond1 0.00492 0.03607 0.01311 0.00820 0.00164 0.01967 0.01967 0.01967

34 Klipheuwel1 0.02131 0.03607 0.02623 0.02131 0.02131 0.00000 0.00000 0.00000

35 Klipheuwel2 0.02131 0.03607 0.02623 0.02131 0.02131 0.00000 0.00000 0.00000

36 Kokstad1 0.03607 0.00328 0.03934 0.03443 0.03770 0.03934 0.03934 0.03934

37 Kokstad2 0.03607 0.00328 0.03934 0.03443 0.03770 0.03934 0.03934 0.03934

38 Kokstad3 0.03443 0.00164 0.03770 0.03279 0.03607 0.03770 0.03770 0.03770

39 Kokstad4 0.03607 0.00328 0.03934 0.03443 0.03770 0.03934 0.03934 0.03934

40 Kokstad5 0.03607 0.00328 0.03934 0.03443 0.03770 0.03934 0.03934 0.03934

41 Kokstad6 0.03279 0.01148 0.03607 0.03115 0.03443 0.03934 0.03934 0.03934

42 Kokstad7 0.03607 0.00328 0.03934 0.03443 0.03770 0.03934 0.03934 0.03934

43 Kraaifontein1 0.02295 0.03443 0.02787 0.02295 0.02295 0.00164 0.00164 0.00164

44 Kwancele1 0.03279 0.01148 0.03607 0.03115 0.03443 0.03934 0.03934 0.03934

45 LakesideCT1 0.00492 0.03607 0.01311 0.00820 0.00164 0.02295 0.02295 0.02295

46 Napier1 0.00164 0.03115 0.01148 0.00656 0.00328 0.02131 0.02131 0.02131

47 Napier2 0.00492 0.03607 0.01311 0.00820 0.00492 0.02295 0.02295 0.02295

48 Napier3 0.00328 0.03443 0.01148 0.00656 0.00328 0.02131 0.02131 0.02131

49 Napier4 0.00656 0.03770 0.01475 0.00984 0.00656 0.02131 0.02131 0.02131

50 Napier5 0.00328 0.03443 0.01148 0.00656 0.00328 0.02131 0.02131 0.02131

51 Napier6 0.00328 0.03443 0.01148 0.00656 0.00328 0.02131 0.02131 0.02131

52 NaturesValley1 0.00984 0.03443 0.00820 0.00656 0.00984 0.02131 0.02131 0.02131

53 Oudtdhoorn1 0.00164 0.03443 0.01311 0.00820 0.00492 0.02295 0.02295 0.02295

54 PortAlfred1 0.00820 0.02951 0.00656 0.00492 0.00820 0.02295 0.02295 0.02295

55 PortStJohns1 0.03279 0.01148 0.03607 0.03115 0.03443 0.03934 0.03934 0.03934

56 PringleBay1 0.00328 0.03443 0.01148 0.00656 0.00000 0.02131 0.02131 0.02131

57 PringleBay2 0.00492 0.03607 0.01311 0.00820 0.00164 0.01967 0.01967 0.01967

58 PringleBay3 0.00328 0.03443 0.01148 0.00656 0.00328 0.02131 0.02131 0.02131

59 PringleBay4 0.00492 0.03607 0.01311 0.00820 0.00164 0.01967 0.01967 0.01967

60 Sabie1 0.03770 0.02459 0.04098 0.03607 0.03934 0.03770 0.03770 0.03770

61 Sabie2 0.03934 0.02623 0.04262 0.03770 0.04098 0.03934 0.03934 0.03934

62 Silvermine1 0.00492 0.03607 0.01311 0.00820 0.00164 0.02295 0.02295 0.02295

63 SomersetWest1 0.02131 0.03607 0.02623 0.02131 0.02131 0.00000 0.00000 0.00000

64 SomersetWest2 0.02131 0.03607 0.02623 0.02131 0.02131 0.00000 0.00000 0.00000

65 SomersetWest3 0.02131 0.03607 0.02623 0.02131 0.02131 0.00000 0.00000 0.00000

66 SomersetWest4 0.02131 0.03607 0.02623 0.02131 0.02131 0.00000 0.00000 0.00000

67 SomersetWest5 0.02131 0.03607 0.02623 0.02131 0.02131 0.00000 0.00000 0.00000

68 SomersetWest6 0.02131 0.03607 0.02623 0.02131 0.02131 0.00000 0.00000 0.00000

69 SomersetWest7 0.02295 0.03770 0.02787 0.02295 0.02295 0.00164 0.00164 0.00164

70 SomersetWest8 0.02131 0.03607 0.02623 0.02131 0.02131 0.00000 0.00000 0.00000

71 Stellenbosch1 0.02131 0.03607 0.02623 0.02131 0.02131 0.00000 0.00000 0.00000

72 Stellenbosch2 0.02131 0.03607 0.02623 0.02131 0.02131 0.00000 0.00000 0.00000

73 Stellenbosch3 0.02131 0.03607 0.02623 0.02131 0.02131 0.00000 0.00000 0.00000

74 Stellenbosch4 0.02131 0.03607 0.02623 0.02131 0.02131 0.00000 0.00000 0.00000

75 Struisbaai1 0.00000 0.03279 0.01148 0.00656 0.00328 0.02131 0.02131 0.02131

76 Swellendam1 0.00328 0.03443 0.01148 0.00656 0.00000 0.02131 0.02131 0.02131

77 Swellendam2 0.00164 0.03115 0.01311 0.00820 0.00492 0.02295 0.02295 0.02295

78 Swellendam3 0.00164 0.03115 0.01311 0.00820 0.00492 0.02295 0.02295 0.02295

79 Swellendam4 0.00000 0.03279 0.01148 0.00656 0.00328 0.02131 0.02131 0.02131

80 Swellendam5 0.00164 0.03115 0.01311 0.00820 0.00492 0.02295 0.02295 0.02295

81 Swellendam6 0.00164 0.03115 0.01311 0.00820 0.00492 0.02295 0.02295 0.02295

82 Swellendam7 0.00164 0.03115 0.01311 0.00820 0.00492 0.02295 0.02295 0.02295

83 Tokai1 0.02131 0.03607 0.02623 0.02131 0.02131 0.00000 0.00000 0.00000

84 Uganda1 0.09672 0.10000 0.09836 0.09672 0.10000 0.10820 0.10820 0.10820

85 Villiersdorp1 0.00328 0.03443 0.01148 0.00656 0.00000 0.02131 0.02131 0.02131

86 Villiersdorp2 0.00328 0.03443 0.01148 0.00656 0.00000 0.02131 0.02131 0.02131

87 Villiersdorp3 0.00328 0.03443 0.01148 0.00656 0.00000 0.02131 0.02131 0.02131

88 Villiersdorp4 0.00328 0.03443 0.01148 0.00656 0.00000 0.02131 0.02131 0.02131

89 Villiersdorp5 0.00328 0.03443 0.01148 0.00656 0.00000 0.02131 0.02131 0.02131

90 Wolkberg1 0.06230 0.06066 0.06557 0.06066 0.06393 0.05902 0.05902 0.05902

91 Kenya1 0.08852 0.08689 0.09016 0.08689 0.08689 0.10164 0.10164 0.10164

92 PortElizabeth 0.00894 0.04022 0.00000 0.00460 0.00663 0.02654 0.02654 0.02654

Supplementary table 2 continues.

33 34 35 36 37 38 39 40

33 Kleinmond1 -

34 Klipheuwel1 0.01967 -

35 Klipheuwel2 0.01967 0.00000 -

36 Kokstad1 0.03934 0.03934 0.03934 -

37 Kokstad2 0.03934 0.03934 0.03934 0.00000 -

38 Kokstad3 0.03770 0.03770 0.03770 0.00164 0.00164 -

39 Kokstad4 0.03934 0.03934 0.03934 0.00000 0.00000 0.00164 -

40 Kokstad5 0.03934 0.03934 0.03934 0.00000 0.00000 0.00164 0.00000 -

41 Kokstad6 0.03607 0.03934 0.03934 0.01148 0.01148 0.00984 0.01148 0.01148

42 Kokstad7 0.03934 0.03934 0.03934 0.00000 0.00000 0.00164 0.00000 0.00000

43 Kraaifontein1 0.02131 0.00164 0.00164 0.03770 0.03770 0.03607 0.03770 0.03770

44 Kwancele1 0.03607 0.03934 0.03934 0.01148 0.01148 0.00984 0.01148 0.01148

45 LakesideCT1 0.00328 0.02295 0.02295 0.03934 0.03934 0.03770 0.03934 0.03934

46 Napier1 0.00492 0.02131 0.02131 0.03443 0.03443 0.03279 0.03443 0.03443

47 Napier2 0.00656 0.02295 0.02295 0.03934 0.03934 0.03770 0.03934 0.03934

48 Napier3 0.00492 0.02131 0.02131 0.03770 0.03770 0.03607 0.03770 0.03770

49 Napier4 0.00820 0.02131 0.02131 0.04098 0.04098 0.03934 0.04098 0.04098

50 Napier5 0.00492 0.02131 0.02131 0.03770 0.03770 0.03607 0.03770 0.03770

51 Napier6 0.00492 0.02131 0.02131 0.03770 0.03770 0.03607 0.03770 0.03770

52 NaturesValley1 0.01148 0.02131 0.02131 0.03770 0.03770 0.03607 0.03770 0.03770

53 Oudtdhoorn1 0.00656 0.02295 0.02295 0.03770 0.03770 0.03607 0.03770 0.03770

54 PortAlfred1 0.00984 0.02295 0.02295 0.03279 0.03279 0.03115 0.03279 0.03279

55 PortStJohns1 0.03607 0.03934 0.03934 0.01148 0.01148 0.00984 0.01148 0.01148

56 PringleBay1 0.00164 0.02131 0.02131 0.03770 0.03770 0.03607 0.03770 0.03770

57 PringleBay2 0.00000 0.01967 0.01967 0.03934 0.03934 0.03770 0.03934 0.03934

58 PringleBay3 0.00492 0.02131 0.02131 0.03770 0.03770 0.03607 0.03770 0.03770

59 PringleBay4 0.00000 0.01967 0.01967 0.03934 0.03934 0.03770 0.03934 0.03934

60 Sabie1 0.04098 0.03770 0.03770 0.02787 0.02787 0.02623 0.02787 0.02787

61 Sabie2 0.04262 0.03934 0.03934 0.02951 0.02951 0.02787 0.02951 0.02951

62 Silvermine1 0.00328 0.02295 0.02295 0.03934 0.03934 0.03770 0.03934 0.03934

63 SomersetWest1 0.01967 0.00000 0.00000 0.03934 0.03934 0.03770 0.03934 0.03934

64 SomersetWest2 0.01967 0.00000 0.00000 0.03934 0.03934 0.03770 0.03934 0.03934

65 SomersetWest3 0.01967 0.00000 0.00000 0.03934 0.03934 0.03770 0.03934 0.03934

66 SomersetWest4 0.01967 0.00000 0.00000 0.03934 0.03934 0.03770 0.03934 0.03934

67 SomersetWest5 0.01967 0.00000 0.00000 0.03934 0.03934 0.03770 0.03934 0.03934

68 SomersetWest6 0.01967 0.00000 0.00000 0.03934 0.03934 0.03770 0.03934 0.03934

69 SomersetWest7 0.02131 0.00164 0.00164 0.04098 0.04098 0.03934 0.04098 0.04098

70 SomersetWest8 0.01967 0.00000 0.00000 0.03934 0.03934 0.03770 0.03934 0.03934

71 Stellenbosch1 0.01967 0.00000 0.00000 0.03934 0.03934 0.03770 0.03934 0.03934

72 Stellenbosch2 0.01967 0.00000 0.00000 0.03934 0.03934 0.03770 0.03934 0.03934

73 Stellenbosch3 0.01967 0.00000 0.00000 0.03934 0.03934 0.03770 0.03934 0.03934

74 Stellenbosch4 0.01967 0.00000 0.00000 0.03934 0.03934 0.03770 0.03934 0.03934

75 Struisbaai1 0.00492 0.02131 0.02131 0.03607 0.03607 0.03443 0.03607 0.03607

76 Swellendam1 0.00164 0.02131 0.02131 0.03770 0.03770 0.03607 0.03770 0.03770

77 Swellendam2 0.00656 0.02295 0.02295 0.03443 0.03443 0.03279 0.03443 0.03443

78 Swellendam3 0.00656 0.02295 0.02295 0.03443 0.03443 0.03279 0.03443 0.03443

79 Swellendam4 0.00492 0.02131 0.02131 0.03607 0.03607 0.03443 0.03607 0.03607

80 Swellendam5 0.00656 0.02295 0.02295 0.03443 0.03443 0.03279 0.03443 0.03443

81 Swellendam6 0.00656 0.02295 0.02295 0.03443 0.03443 0.03279 0.03443 0.03443

82 Swellendam7 0.00656 0.02295 0.02295 0.03443 0.03443 0.03279 0.03443 0.03443

83 Tokai1 0.01967 0.00000 0.00000 0.03934 0.03934 0.03770 0.03934 0.03934

84 Uganda1 0.10164 0.10820 0.10820 0.10000 0.10000 0.10164 0.10000 0.10000

85 Villiersdorp1 0.00164 0.02131 0.02131 0.03770 0.03770 0.03607 0.03770 0.03770

86 Villiersdorp2 0.00164 0.02131 0.02131 0.03770 0.03770 0.03607 0.03770 0.03770

87 Villiersdorp3 0.00164 0.02131 0.02131 0.03770 0.03770 0.03607 0.03770 0.03770

88 Villiersdorp4 0.00164 0.02131 0.02131 0.03770 0.03770 0.03607 0.03770 0.03770

89 Villiersdorp5 0.00164 0.02131 0.02131 0.03770 0.03770 0.03607 0.03770 0.03770

90 Wolkberg1 0.06230 0.05902 0.05902 0.06393 0.06393 0.06230 0.06393 0.06393

91 Kenya1 0.08852 0.10164 0.10164 0.08689 0.08689 0.08852 0.08689 0.08689

92 PortElizabeth 0.00889 0.02654 0.02654 0.04226 0.04226 0.04224 0.04226 0.04226

Supplementary table 2 continues.

41 42 43 44 45 46 47 48

41 Kokstad6 -

42 Kokstad7 0.01148 -

43 Kraaifontein1 0.03770 0.03770 -

44 Kwancele1 0.00000 0.01148 0.03770 -

45 LakesideCT1 0.03607 0.03934 0.02459 0.03607 -

46 Napier1 0.03115 0.03443 0.02295 0.03115 0.00492 -

47 Napier2 0.03607 0.03934 0.02459 0.03607 0.00656 0.00492 -

48 Napier3 0.03443 0.03770 0.02295 0.03443 0.00492 0.00328 0.00164 -

49 Napier4 0.03770 0.04098 0.02295 0.03770 0.00820 0.00656 0.00492 0.00328

50 Napier5 0.03443 0.03770 0.02295 0.03443 0.00492 0.00328 0.00164 0.00000

51 Napier6 0.03443 0.03770 0.02295 0.03443 0.00492 0.00328 0.00164 0.00000

52 NaturesValley1 0.03443 0.03770 0.02295 0.03443 0.01148 0.00984 0.01148 0.00984

53 Oudtdhoorn1 0.03443 0.03770 0.02459 0.03443 0.00656 0.00328 0.00656 0.00492

54 PortAlfred1 0.02951 0.03279 0.02459 0.02951 0.00984 0.00820 0.00984 0.00820

55 PortStJohns1 0.00000 0.01148 0.03770 0.00000 0.03607 0.03115 0.03607 0.03443

56 PringleBay1 0.03443 0.03770 0.02295 0.03443 0.00164 0.00328 0.00492 0.00328

57 PringleBay2 0.03607 0.03934 0.02131 0.03607 0.00328 0.00492 0.00656 0.00492

58 PringleBay3 0.03443 0.03770 0.02295 0.03443 0.00492 0.00328 0.00164 0.00000

59 PringleBay4 0.03607 0.03934 0.02131 0.03607 0.00328 0.00492 0.00656 0.00492

60 Sabie1 0.02787 0.02787 0.03934 0.02787 0.04098 0.03607 0.04098 0.03934

61 Sabie2 0.02951 0.02951 0.04098 0.02951 0.04262 0.03770 0.04262 0.04098

62 Silvermine1 0.03607 0.03934 0.02459 0.03607 0.00328 0.00492 0.00656 0.00492

63 SomersetWest1 0.03934 0.03934 0.00164 0.03934 0.02295 0.02131 0.02295 0.02131

64 SomersetWest2 0.03934 0.03934 0.00164 0.03934 0.02295 0.02131 0.02295 0.02131

65 SomersetWest3 0.03934 0.03934 0.00164 0.03934 0.02295 0.02131 0.02295 0.02131

66 SomersetWest4 0.03934 0.03934 0.00164 0.03934 0.02295 0.02131 0.02295 0.02131

67 SomersetWest5 0.03934 0.03934 0.00164 0.03934 0.02295 0.02131 0.02295 0.02131

68 SomersetWest6 0.03934 0.03934 0.00164 0.03934 0.02295 0.02131 0.02295 0.02131

69 SomersetWest7 0.04098 0.04098 0.00328 0.04098 0.02459 0.02295 0.02459 0.02295

70 SomersetWest8 0.03934 0.03934 0.00164 0.03934 0.02295 0.02131 0.02295 0.02131

71 Stellenbosch1 0.03934 0.03934 0.00164 0.03934 0.02295 0.02131 0.02295 0.02131

72 Stellenbosch2 0.03934 0.03934 0.00164 0.03934 0.02295 0.02131 0.02295 0.02131

73 Stellenbosch3 0.03934 0.03934 0.00164 0.03934 0.02295 0.02131 0.02295 0.02131

74 Stellenbosch4 0.03934 0.03934 0.00164 0.03934 0.02295 0.02131 0.02295 0.02131

75 Struisbaai1 0.03279 0.03607 0.02295 0.03279 0.00492 0.00164 0.00492 0.00328

76 Swellendam1 0.03443 0.03770 0.02295 0.03443 0.00164 0.00328 0.00492 0.00328

77 Swellendam2 0.03115 0.03443 0.02459 0.03115 0.00656 0.00328 0.00656 0.00492

78 Swellendam3 0.03115 0.03443 0.02459 0.03115 0.00656 0.00328 0.00656 0.00492

79 Swellendam4 0.03279 0.03607 0.02295 0.03279 0.00492 0.00164 0.00492 0.00328

80 Swellendam5 0.03115 0.03443 0.02459 0.03115 0.00656 0.00328 0.00656 0.00492

81 Swellendam6 0.03115 0.03443 0.02459 0.03115 0.00656 0.00328 0.00656 0.00492

82 Swellendam7 0.03115 0.03443 0.02459 0.03115 0.00656 0.00328 0.00656 0.00492

83 Tokai1 0.03934 0.03934 0.00164 0.03934 0.02295 0.02131 0.02295 0.02131

84 Uganda1 0.10164 0.10000 0.10656 0.10164 0.10164 0.09836 0.09836 0.10000

85 Villiersdorp1 0.03443 0.03770 0.02295 0.03443 0.00164 0.00328 0.00492 0.00328

86 Villiersdorp2 0.03443 0.03770 0.02295 0.03443 0.00164 0.00328 0.00492 0.00328

87 Villiersdorp3 0.03443 0.03770 0.02295 0.03443 0.00164 0.00328 0.00492 0.00328

88 Villiersdorp4 0.03443 0.03770 0.02295 0.03443 0.00164 0.00328 0.00492 0.00328

89 Villiersdorp5 0.03443 0.03770 0.02295 0.03443 0.00164 0.00328 0.00492 0.00328

90 Wolkberg1 0.06230 0.06393 0.06066 0.06230 0.06557 0.06066 0.06557 0.06393

91 Kenya1 0.08852 0.08689 0.10000 0.08852 0.08852 0.08852 0.09180 0.09016

92 PortElizabeth 0.04017 0.04226 0.02654 0.04017 0.00663 0.00889 0.01118 0.00889

Supplementary table 2 continues.

49 50 51 52 53 54 55 56

49 Napier4 -

50 Napier5 0.00328 -

51 Napier6 0.00328 0.00000 -

52 NaturesValley1 0.01311 0.00984 0.00984 -

53 Oudtdhoorn1 0.00820 0.00492 0.00492 0.01148 -

54 PortAlfred1 0.01148 0.00820 0.00820 0.00820 0.00984 -

55 PortStJohns1 0.03770 0.03443 0.03443 0.03443 0.03443 0.02951 -

56 PringleBay1 0.00656 0.00328 0.00328 0.00984 0.00492 0.00820 0.03443 -

57 PringleBay2 0.00820 0.00492 0.00492 0.01148 0.00656 0.00984 0.03607 0.00164

58 PringleBay3 0.00328 0.00000 0.00000 0.00984 0.00492 0.00820 0.03443 0.00328

59 PringleBay4 0.00820 0.00492 0.00492 0.01148 0.00656 0.00984 0.03607 0.00164

60 Sabie1 0.03934 0.03934 0.03934 0.03934 0.03934 0.03443 0.02787 0.03934

61 Sabie2 0.04098 0.04098 0.04098 0.04098 0.04098 0.03607 0.02951 0.04098

62 Silvermine1 0.00820 0.00492 0.00492 0.01148 0.00656 0.00984 0.03607 0.00164

63 SomersetWest1 0.02131 0.02131 0.02131 0.02131 0.02295 0.02295 0.03934 0.02131

64 SomersetWest2 0.02131 0.02131 0.02131 0.02131 0.02295 0.02295 0.03934 0.02131

65 SomersetWest3 0.02131 0.02131 0.02131 0.02131 0.02295 0.02295 0.03934 0.02131

66 SomersetWest4 0.02131 0.02131 0.02131 0.02131 0.02295 0.02295 0.03934 0.02131

67 SomersetWest5 0.02131 0.02131 0.02131 0.02131 0.02295 0.02295 0.03934 0.02131

68 SomersetWest6 0.02131 0.02131 0.02131 0.02131 0.02295 0.02295 0.03934 0.02131

69 SomersetWest7 0.02295 0.02295 0.02295 0.02295 0.02459 0.02459 0.04098 0.02295

70 SomersetWest8 0.02131 0.02131 0.02131 0.02131 0.02295 0.02295 0.03934 0.02131

71 Stellenbosch1 0.02131 0.02131 0.02131 0.02131 0.02295 0.02295 0.03934 0.02131

72 Stellenbosch2 0.02131 0.02131 0.02131 0.02131 0.02295 0.02295 0.03934 0.02131

73 Stellenbosch3 0.02131 0.02131 0.02131 0.02131 0.02295 0.02295 0.03934 0.02131

74 Stellenbosch4 0.02131 0.02131 0.02131 0.02131 0.02295 0.02295 0.03934 0.02131

75 Struisbaai1 0.00656 0.00328 0.00328 0.00984 0.00164 0.00820 0.03279 0.00328

76 Swellendam1 0.00656 0.00328 0.00328 0.00984 0.00492 0.00820 0.03443 0.00000

77 Swellendam2 0.00820 0.00492 0.00492 0.01148 0.00328 0.00984 0.03115 0.00492

78 Swellendam3 0.00820 0.00492 0.00492 0.01148 0.00328 0.00984 0.03115 0.00492

79 Swellendam4 0.00656 0.00328 0.00328 0.00984 0.00164 0.00820 0.03279 0.00328

80 Swellendam5 0.00820 0.00492 0.00492 0.01148 0.00328 0.00984 0.03115 0.00492

81 Swellendam6 0.00820 0.00492 0.00492 0.01148 0.00328 0.00984 0.03115 0.00492

82 Swellendam7 0.00820 0.00492 0.00492 0.01148 0.00328 0.00984 0.03115 0.00492

83 Tokai1 0.02131 0.02131 0.02131 0.02131 0.02295 0.02295 0.03934 0.02131

84 Uganda1 0.10000 0.10000 0.10000 0.10000 0.09672 0.09508 0.10164 0.10000

85 Villiersdorp1 0.00656 0.00328 0.00328 0.00984 0.00492 0.00820 0.03443 0.00000

86 Villiersdorp2 0.00656 0.00328 0.00328 0.00984 0.00492 0.00820 0.03443 0.00000

87 Villiersdorp3 0.00656 0.00328 0.00328 0.00984 0.00492 0.00820 0.03443 0.00000

88 Villiersdorp4 0.00656 0.00328 0.00328 0.00984 0.00492 0.00820 0.03443 0.00000

89 Villiersdorp5 0.00656 0.00328 0.00328 0.00984 0.00492 0.00820 0.03443 0.00000

90 Wolkberg1 0.06393 0.06393 0.06393 0.06393 0.06393 0.05902 0.06230 0.06393

91 Kenya1 0.09016 0.09016 0.09016 0.09180 0.08852 0.08361 0.08852 0.08689

92 PortElizabeth 0.01114 0.00889 0.00889 0.00888 0.01123 0.00460 0.04017 0.00663

Supplementary table 2 continues.

57 58 59 60 61 62 63 64

57 PringleBay2 -

58 PringleBay3 0.00492 -

59 PringleBay4 0.00000 0.00492 -

60 Sabie1 0.04098 0.03934 0.04098 -

61 Sabie2 0.04262 0.04098 0.04262 0.00164 -

62 Silvermine1 0.00328 0.00492 0.00328 0.04098 0.04262 -

63 SomersetWest1 0.01967 0.02131 0.01967 0.03770 0.03934 0.02295 -

64 SomersetWest2 0.01967 0.02131 0.01967 0.03770 0.03934 0.02295 0.00000 -

65 SomersetWest3 0.01967 0.02131 0.01967 0.03770 0.03934 0.02295 0.00000 0.00000

66 SomersetWest4 0.01967 0.02131 0.01967 0.03770 0.03934 0.02295 0.00000 0.00000

67 SomersetWest5 0.01967 0.02131 0.01967 0.03770 0.03934 0.02295 0.00000 0.00000

68 SomersetWest6 0.01967 0.02131 0.01967 0.03770 0.03934 0.02295 0.00000 0.00000

69 SomersetWest7 0.02131 0.02295 0.02131 0.03934 0.04098 0.02459 0.00164 0.00164

70 SomersetWest8 0.01967 0.02131 0.01967 0.03770 0.03934 0.02295 0.00000 0.00000

71 Stellenbosch1 0.01967 0.02131 0.01967 0.03770 0.03934 0.02295 0.00000 0.00000

72 Stellenbosch2 0.01967 0.02131 0.01967 0.03770 0.03934 0.02295 0.00000 0.00000

73 Stellenbosch3 0.01967 0.02131 0.01967 0.03770 0.03934 0.02295 0.00000 0.00000

74 Stellenbosch4 0.01967 0.02131 0.01967 0.03770 0.03934 0.02295 0.00000 0.00000

75 Struisbaai1 0.00492 0.00328 0.00492 0.03770 0.03934 0.00492 0.02131 0.02131

76 Swellendam1 0.00164 0.00328 0.00164 0.03934 0.04098 0.00164 0.02131 0.02131

77 Swellendam2 0.00656 0.00492 0.00656 0.03607 0.03770 0.00656 0.02295 0.02295

78 Swellendam3 0.00656 0.00492 0.00656 0.03607 0.03770 0.00656 0.02295 0.02295

79 Swellendam4 0.00492 0.00328 0.00492 0.03770 0.03934 0.00492 0.02131 0.02131

80 Swellendam5 0.00656 0.00492 0.00656 0.03607 0.03770 0.00656 0.02295 0.02295

81 Swellendam6 0.00656 0.00492 0.00656 0.03607 0.03770 0.00656 0.02295 0.02295

82 Swellendam7 0.00656 0.00492 0.00656 0.03607 0.03770 0.00656 0.02295 0.02295

83 Tokai1 0.01967 0.02131 0.01967 0.03770 0.03934 0.02295 0.00000 0.00000

84 Uganda1 0.10164 0.10000 0.10164 0.09508 0.09672 0.09836 0.10820 0.10820

85 Villiersdorp1 0.00164 0.00328 0.00164 0.03934 0.04098 0.00164 0.02131 0.02131

86 Villiersdorp2 0.00164 0.00328 0.00164 0.03934 0.04098 0.00164 0.02131 0.02131

87 Villiersdorp3 0.00164 0.00328 0.00164 0.03934 0.04098 0.00164 0.02131 0.02131

88 Villiersdorp4 0.00164 0.00328 0.00164 0.03934 0.04098 0.00164 0.02131 0.02131

89 Villiersdorp5 0.00164 0.00328 0.00164 0.03934 0.04098 0.00164 0.02131 0.02131

90 Wolkberg1 0.06230 0.06393 0.06230 0.06393 0.06557 0.06557 0.05902 0.05902

91 Kenya1 0.08852 0.09016 0.08852 0.09016 0.09180 0.08525 0.10164 0.10164

92 PortElizabeth 0.00889 0.00889 0.00889 0.04924 0.04921 0.00663 0.02654 0.02654

Supplementary table 2 continues.

65 66 67 68 69 70 71 72

65 SomersetWest3 -

66 SomersetWest4 0.00000 -

67 SomersetWest5 0.00000 0.00000 -

68 SomersetWest6 0.00000 0.00000 0.00000 -

69 SomersetWest7 0.00164 0.00164 0.00164 0.00164 -

70 SomersetWest8 0.00000 0.00000 0.00000 0.00000 0.00164 -

71 Stellenbosch1 0.00000 0.00000 0.00000 0.00000 0.00164 0.00000 -

72 Stellenbosch2 0.00000 0.00000 0.00000 0.00000 0.00164 0.00000 0.00000 -

73 Stellenbosch3 0.00000 0.00000 0.00000 0.00000 0.00164 0.00000 0.00000 0.00000

74 Stellenbosch4 0.00000 0.00000 0.00000 0.00000 0.00164 0.00000 0.00000 0.00000

75 Struisbaai1 0.02131 0.02131 0.02131 0.02131 0.02295 0.02131 0.02131 0.02131

76 Swellendam1 0.02131 0.02131 0.02131 0.02131 0.02295 0.02131 0.02131 0.02131

77 Swellendam2 0.02295 0.02295 0.02295 0.02295 0.02459 0.02295 0.02295 0.02295

78 Swellendam3 0.02295 0.02295 0.02295 0.02295 0.02459 0.02295 0.02295 0.02295

79 Swellendam4 0.02131 0.02131 0.02131 0.02131 0.02295 0.02131 0.02131 0.02131

80 Swellendam5 0.02295 0.02295 0.02295 0.02295 0.02459 0.02295 0.02295 0.02295

81 Swellendam6 0.02295 0.02295 0.02295 0.02295 0.02459 0.02295 0.02295 0.02295

82 Swellendam7 0.02295 0.02295 0.02295 0.02295 0.02459 0.02295 0.02295 0.02295

83 Tokai1 0.00000 0.00000 0.00000 0.00000 0.00164 0.00000 0.00000 0.00000

84 Uganda1 0.10820 0.10820 0.10820 0.10820 0.10984 0.10820 0.10820 0.10820

85 Villiersdorp1 0.02131 0.02131 0.02131 0.02131 0.02295 0.02131 0.02131 0.02131

86 Villiersdorp2 0.02131 0.02131 0.02131 0.02131 0.02295 0.02131 0.02131 0.02131

87 Villiersdorp3 0.02131 0.02131 0.02131 0.02131 0.02295 0.02131 0.02131 0.02131

88 Villiersdorp4 0.02131 0.02131 0.02131 0.02131 0.02295 0.02131 0.02131 0.02131

89 Villiersdorp5 0.02131 0.02131 0.02131 0.02131 0.02295 0.02131 0.02131 0.02131

90 Wolkberg1 0.05902 0.05902 0.05902 0.05902 0.06066 0.05902 0.05902 0.05902

91 Kenya1 0.10164 0.10164 0.10164 0.10164 0.10328 0.10164 0.10164 0.10164

92 PortElizabeth 0.02654 0.02654 0.02654 0.02654 0.02855 0.02654 0.02654 0.02654

Supplementary table 2 continues.

73 74 75 76 77 78 79 80

73 Stellenbosch3 -

74 Stellenbosch4 0.00000 -

75 Struisbaai1 0.02131 0.02131 -

76 Swellendam1 0.02131 0.02131 0.00328 -

77 Swellendam2 0.02295 0.02295 0.00164 0.00492 -

78 Swellendam3 0.02295 0.02295 0.00164 0.00492 0.00000 -

79 Swellendam4 0.02131 0.02131 0.00000 0.00328 0.00164 0.00164 -

80 Swellendam5 0.02295 0.02295 0.00164 0.00492 0.00000 0.00000 0.00164 -

81 Swellendam6 0.02295 0.02295 0.00164 0.00492 0.00000 0.00000 0.00164 0.00000

82 Swellendam7 0.02295 0.02295 0.00164 0.00492 0.00000 0.00000 0.00164 0.00000

83 Tokai1 0.00000 0.00000 0.02131 0.02131 0.02295 0.02295 0.02131 0.02295

84 Uganda1 0.10820 0.10820 0.09672 0.10000 0.09508 0.09508 0.09672 0.09508

85 Villiersdorp1 0.02131 0.02131 0.00328 0.00000 0.00492 0.00492 0.00328 0.00492

86 Villiersdorp2 0.02131 0.02131 0.00328 0.00000 0.00492 0.00492 0.00328 0.00492

87 Villiersdorp3 0.02131 0.02131 0.00328 0.00000 0.00492 0.00492 0.00328 0.00492

88 Villiersdorp4 0.02131 0.02131 0.00328 0.00000 0.00492 0.00492 0.00328 0.00492

89 Villiersdorp5 0.02131 0.02131 0.00328 0.00000 0.00492 0.00492 0.00328 0.00492

90 Wolkberg1 0.05902 0.05902 0.06230 0.06393 0.06393 0.06393 0.06230 0.06393

91 Kenya1 0.10164 0.10164 0.08852 0.08689 0.08689 0.08689 0.08852 0.08689

92 PortElizabeth 0.02654 0.02654 0.00894 0.00663 0.01123 0.01123 0.00894 0.01123

Uncorrected ("p") distance matrix (continued)

81 82 83 84 85 86 87 88

81 Swellendam6 -

82 Swellendam7 0.00000 -

83 Tokai1 0.02295 0.02295 -

84 Uganda1 0.09508 0.09508 0.10820 -

85 Villiersdorp1 0.00492 0.00492 0.02131 0.10000 -

86 Villiersdorp2 0.00492 0.00492 0.02131 0.10000 0.00000 -

87 Villiersdorp3 0.00492 0.00492 0.02131 0.10000 0.00000 0.00000 -

88 Villiersdorp4 0.00492 0.00492 0.02131 0.10000 0.00000 0.00000 0.00000 -

89 Villiersdorp5 0.00492 0.00492 0.02131 0.10000 0.00000 0.00000 0.00000 0.00000

90 Wolkberg1 0.06393 0.06393 0.05902 0.11311 0.06393 0.06393 0.06393 0.06393

91 Kenya1 0.08689 0.08689 0.10164 0.04590 0.08689 0.08689 0.08689 0.08689

92 PortElizabeth 0.01123 0.01123 0.02654 0.10462 0.00663 0.00663 0.00663 0.00663

Supplementary table 2 continues.

89 90 91 92

89 Villiersdorp5 -

90 Wolkberg1 0.06393 -

91 Kenya1 0.08689 0.10328 -

92 PortElizabeth 0.00663 0.06243 0.09373 -
