# Supplementary material for: Molecular evidence for cryptic species in the common slug eating snake Duberrialutrixlutrix (Squamata, Lamprophiidae) from South Africa
Source: Zookeys. 2019 Apr 15;838:133–54. doi: 10.3897/zookeys.838.32022 (PMC6477839; doi:10.3897/zookeys.838.32022)
Supplement: Supplementary material 3 [file zookeys-838-133-s003.docx]

**Supplementary table 3.** Distribution of the 35 haplotype frequency for the combined mtDNA loci sequence data for the 87 *Duberria lutrix lutrix* specimens. The haplotype numbers (N) corresponds to the numbers on the minimum spanning network (Fig. 3).

| Locality | Haplotype N | |  |  |  |  |  |  |  |  |  |
| --- | --- | --- | --- | --- | --- | --- | --- | --- | --- | --- | --- |
|  | 1 | 2 | 3 | 4 | 5 | 6 | 7 | 8 | 9 | 10 | 11 |
| Ashton | 8 |  |  |  |  |  |  |  |  |  |  |
| Bredasdorp |  | 1 |  |  |  |  |  |  |  |  |  |
| Herbertsdale |  |  | 1 |  |  |  |  |  |  |  |  |
| Struisbaai |  | 1 |  |  |  |  |  |  |  |  |  |
| Swellendam |  | 1 |  | 5 |  |  |  |  |  |  |  |
| Agulhas |  |  |  | 1 |  |  |  |  |  |  |  |
| Natures Valley |  |  |  |  | 1 |  |  |  |  |  |  |
| Grahamstown |  |  |  |  |  | 1 |  |  |  |  |  |
| Port Elizabeth |  |  |  |  |  | 1 |  |  |  |  |  |
| Hope Fountain |  |  |  |  |  | 1 |  |  |  |  |  |
| Port Alfred |  |  |  |  |  |  | 1 |  |  |  |  |
| Humansdorp |  |  |  |  |  |  |  | 1 |  |  |  |
| Lakeside |  |  |  |  |  |  |  |  | 1 |  |  |
| Kleinmond |  |  |  |  |  |  |  |  |  | 1 |  |
| Pringle Bay |  |  |  |  |  |  |  |  |  | 2 | 1 |

**Supplementary table 3.** continues.

|  | Haplotype N | |  |  |  |  |  |  |  |  |  |
| --- | --- | --- | --- | --- | --- | --- | --- | --- | --- | --- | --- |
| Locality | 12 | 13 | 14 | 15 | 16 | 17 | 18 | 19 | 20 | 21 | 22 |
| Silvermine | 1 |  |  |  |  |  |  |  |  |  |  |
| Genadendal |  | 1 |  |  |  |  |  |  |  |  |  |
| Greyton |  | 3 |  |  |  |  |  |  |  |  |  |
| Swellendam |  | 1 |  |  |  |  |  |  |  |  |  |
| Jacobs Bay |  | 1 |  |  |  |  |  |  |  |  |  |
| Villiersdorp |  | 5 |  |  |  |  |  |  |  |  |  |
| Napier |  |  | 1 | 2 | 1 | 1 | 1 |  |  |  |  |
| Caledon |  |  |  | 1 |  |  |  |  |  |  |  |
| Pringle Bay |  |  |  | 1 |  |  |  |  |  |  |  |
| Agulhas |  |  |  |  |  |  |  | 1 |  |  |  |
| Oudtshoorn |  |  |  |  |  |  |  |  | 1 |  |  |
| Flakkenberg |  |  |  |  |  |  |  |  |  | 1 |  |
| Kirstenbosch |  |  |  |  |  |  |  |  |  | 3 |  |
| Somerset West |  |  |  |  |  |  |  |  |  | 3 | 1 |
| Tokai |  |  |  |  |  |  |  |  |  | 1 |  |

**Supplementary table 3.** continues.

|  | Haplotype N | |  |  |  |  |  |  |  |  |  |
| --- | --- | --- | --- | --- | --- | --- | --- | --- | --- | --- | --- |
| Locality | 23 | 24 | 25 | 26 | 27 | 28 | 29 | 30 | 31 | 32 | 33 |
| Kraaifontein | 1 |  |  |  |  |  |  |  |  |  |  |
| Klipheuwel |  | 1 |  | 1 |  |  |  |  |  |  |  |
| Somerset West |  | 1 | 1 | 2 |  |  |  |  |  |  |  |
| Bergvliet |  |  |  | 1 |  |  |  |  |  |  |  |
| Stellenbosch |  |  |  | 4 |  |  |  |  |  |  |  |
| Kokstad |  |  |  |  | 5 |  | 1 |  | 1 |  |  |
| High Water |  |  |  |  |  | 1 |  |  |  |  |  |
| Kwancele |  |  |  |  |  |  |  | 1 |  |  |  |
| Port StJohns |  |  |  |  |  |  |  |  | 1 |  |  |
| Sabie |  |  |  |  |  |  |  |  |  | 1 | 1 |

|  | Haplotype N | |
| --- | --- | --- |
| Locality | 34 | 35 |
| Wolkberg | 1 |  |
| Entabeni |  | 1 |
